# Multifunctional Nacre-Like Nanocomposite Papers for Electromagnetic Interference Shielding via Heterocyclic Aramid/MXene Template-Assisted *In-Situ* Polypyrrole Assembly

**DOI:** 10.1007/s40820-024-01552-9

**Published:** 2024-10-31

**Authors:** Jinhua Xiong, Xu Zhao, Zonglin Liu, He Chen, Qian Yan, Huanxin Lian, Yunxiang Chen, Qingyu Peng, Xiaodong He

**Affiliations:** National Key Laboratory of Science and Technology On Advanced Composites in Special Environments, Center for Composite Materials and Structures, Harbin Institute of Technology, Harbin, 150080 People’s Republic of China

**Keywords:** MXene, Remarkable mechanical properties, Heterocyclic aramid, Electromagnetic interference shielding, Polypyrrole, Multifunctionality

## Abstract

Robust, ultra-flexible, and multifunctional MXene-based electromagnetic interference (EMI) shielding nanocomposite films exhibit enormous potential for applications in artificial intelligence, wireless telecommunication, and portable/wearable electronic equipment. In this work, a nacre-inspired multifunctional heterocyclic aramid (HA)/MXene@polypyrrole (PPy) (HMP) nanocomposite paper with large-scale, high strength, super toughness, and excellent tolerance to complex conditions is fabricated through the strategy of HA/MXene hydrogel template-assisted *in-situ* assembly of PPy. Benefiting from the "brick-and-mortar" layered structure and the strong hydrogen-bonding interactions among MXene, HA, and PPy, the paper exhibits remarkable mechanical performances, including high tensile strength (309.7 MPa), outstanding toughness (57.6 MJ m^−3^), exceptional foldability, and structural stability against ultrasonication. By using the template effect of HA/MXene to guide the assembly of conductive polymers, the synthesized paper obtains excellent electronic conductivity. More importantly, the highly continuous conductive path enables the nanocomposite paper to achieve a splendid EMI shielding effectiveness (EMI SE) of 54.1 dB at an ultra-thin thickness (25.4 μm) and a high specific EMI SE of 17,204.7 dB cm^2^ g^−1^. In addition, the papers also have excellent applications in electromagnetic protection, electro-/photothermal de-icing, thermal therapy, and fire safety. These findings broaden the ideas for developing high-performance and multifunctional MXene-based films with enormous application potential in EMI shielding and thermal management.

**Supplementary Information:**

The online version contains supplementary material available at 10.1007/s40820-024-01552-9.

## Highlights

- The large-scale, high-strength, super-tough, and multifunctional nacre-like heterocyclic aramid (HA)/MXene@polypyrrole (PPy) (HMP) nanocomposite papers were fabricated using the *in-situ* assembly of PPy onto the HA/MXene hydrogel template.
- The "brick-and-mortar" layered structure and abundant hydrogen-bonding interactions among MXene, PPy, and HA respond cooperatively to external stress and effectively increase the mechanical properties of HMP nanocomposite papers.
- The templating effect from HA/MXene was utilized to guide the assembly of conducting polymers, leading to high electrical conductivity and outstanding electromagnetic interference shielding performance.

**Supplementary Information:**

The online version contains supplementary material available at 10.1007/s40820-024-01552-9.

## Graphical abstract

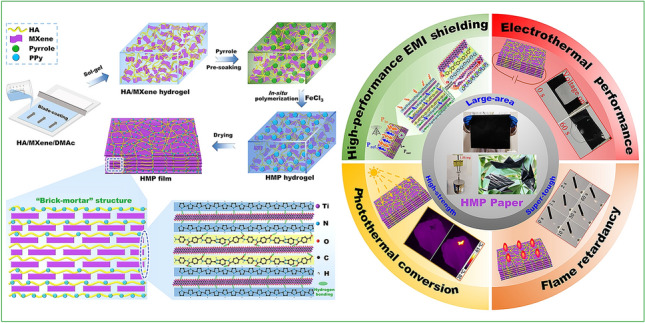

## Introduction

The rapid advancement of wireless telecommunication requires the development of high-performance electromagnetic interference (EMI) shielding materials to ensure the stable operation of precision equipment and prevent the human body from being harmed by electromagnetic radiation [1–3]. In particular, with the growing requirement for EMI shielding material for flexible electronic devices, there is an even more urgent need for next-generation ultra-flexible shielding materials with comprehensive properties such as lightweight, high strength, super toughness, excellent durability, intelligent heating, and thermal stability [4, 5]. As an emerging graphene-like two-dimensional nanomaterial, Ti_3_C_2_T_x_ MXene is a promising EMI shielding material thanks to its ultra-high conductivity, abundant surface functional groups, and accessible film-forming properties [6, 7]. For example, Shahzad and co-workers prepared an 11.2-μm-thick free-standing MXene film using vacuum-assisted filtration, which achieved excellent shielding effectiveness (SE) of 68 dB [8]. Nevertheless, pure MXene films face problems such as inevitable oxidation under O_2_/H_2_O conditions and poor mechanical properties due to weak interactions between MXene nanosheets, which seriously hinders their applications [9, 10].

Excellent mechanical properties are of great value for the design and development of high-performance EMI shielding materials that can endure various complex flexible deformations, especially in communication applications, aerospace, and wearable electronic devices [11, 12]. In recent years, the construction of nacre-like "brick-and-mortar" structures and strong hydrogen bond interaction in MXene/polymer composites have been used to enhance the mechanical performances of MXene-based macrostructures [13]. For example, Cao et al. used a facile vacuum filtration strategy to construct a nacre-inspired MXene/cellulose nanofiber nanocomposite film for EMI shielding with remarkable mechanical properties (tensile strength: 135.4 MPa, strain: 16.7%) and satisfactory EMI SE value of 24.0 dB at an ultra-thin thickness of 47 μm [14]. Sun and colleagues fabricated a nacre-mimetic MXene-xanthan film with strong tensile strength (116.48 MPa) and splendid specific EMI SE (14,490.1 dB cm^2^ g^−1^) [15]. Despite these encouraging advances, these polymers' relatively low inherent mechanical properties and weak interfacial binding with MXene hinder practical use in many fields requiring outstanding mechanical performances. In addition, most current polymer-based composite films still face the problems of high-temperature softening, low-temperature brittleness, weak thermal-shock resistance, and ignitability, which severely limit practical applications under complex and extreme conditions [16, 17]. Thus, the development of MXene-based EMI shielding materials that simultaneously possess excellent mechanical properties and outstanding functionality and endure extreme environments remains a difficult task.

Recently, aromatic polyamides such as aramid nanofiber (ANF) and heterocyclic aramid (HA), a class of polymers with intrinsically remarkable mechanical performances, high-temperature stability, and flame retardancy, have been applied in EMI shielding films combined with two-dimensional layered materials such as MXene and graphene [18, 19]. In particular, nacre-mimic aramid/MXene composite films have made good progress in the field of EMI shielding. For instance, Wang and co-workers reported that nacre-like MXene/ANF composite films fabricated by sol-gel-film technique with remarkable mechanical properties (210.8 MPa, 8.8 MJ m^−3^), a splendid specific EMI SE of 8008.4 dB cm^2^ g^−1^, and excellent electrothermal conversion property [20]. Xiong et al. prepared a nacre-inspired MXene/HA film with strong tensile strength (203.25 MPa) and outstanding specific EMI SE (14,529.1 dB cm^2^ g^−1^) [21]. Although these insulating aromatic polyamides typically penetrate the MXene interlayers as binders to enhance the mechanical performances of the resulting nacre-mimic MXene/aramid nanocomposite films, the conductivity inevitably deteriorates. In general, high MXene content (above 50%) is usually required to overcome the insulating polymer barrier between MXene nanosheets to improve conductivity and obtain satisfactory EMI SE, but its mechanical properties will be seriously affected. Therefore, conductive polymers such as polypyrrole (PPy) [22], polyaniline (PANI) [23], poly(3,4-ethylenedioxythiophene): poly(styrene sulfonate) (PEDOT: PSS) [24] have been used as alternatives to insulating polymers in MXene-polymer composite EMI materials in recent years thanks to their remarkable conductivity. For example, Liu et al. designed and fabricated a flexible free-standing PEDOT: PSS/MXene nanocomposite film with a nacre-mimetic layered structure by vacuum-assisted filtration [25]. The film displayed an ultrahigh electrical conductivity of 34,050 S m^−1^ and a brilliant specific EMI SE of 19,497.8 dB cm^2^ g^−1^. Although these results are exciting, these MXene/polymer composite films have shown an improvement in either mechanical properties or EMI shielding performance or functionalities while sacrificing one property over the other. Thus, to simultaneously enhance the EMI SE, mechanical properties, and various functions of high-performance EMI shielding materials, it is necessary to combine reasonable material selection, effective microstructure design, and interaction between organic and inorganic interfaces [12]. Furthermore, since the size of the filtration equipment influences different manufacturing batches and film performances, the above laboratory-scale vacuum-assisted filtration is challenging in the realization of actual large-scale production [20]. In short, a facile, high-efficiency, scalable fabrication of high-strength, ultra-flexible, durable MXene-based EMI shielding films with high-performance and efficient multifunctionality remains hugely challenging, especially in finding a compromise between these properties that does not come at the expense of the other.

To solve the abovementioned problems, the HA/MXene composite hydrogel templates were first produced using the blade-coating method and sol-gel transformation approach. Then, the large-area, robust, ultratough, and multifunctional nacre-like HA/MXene@PPy (HMP) nanocomposite papers were prepared by the *in-situ* assembly of PPy onto the HA/MXene templates. In this scheme, the nacre-mimetic layered structure and strong hydrogen-bonding effect among MXene, PPy, and HA synergistically responded to external stress, significantly improving the mechanical performances of HMP paper. Therefore, the HMP nanocomposite paper displays enhanced mechanical performances with a tensile strength of 309.7 MPa and a fracture toughness of 57.6 MJ m^−3^ and meanwhile demonstrates outstanding folding resistance (almost no decrease in mechanical properties after repeated folding 10,000 times) and structural stability (no damage after 3 h of ultrasound). Furthermore, the templating effect from HA/MXene is utilized to guide the assembly of conducting polymers, leading to excellent electrical conductivity and brilliant EMI SE values. Surprisingly, the paper also presents remarkable electro-/photothermal performance, thermal stability, and fire resistance. Combined with the simple and efficient manufacturing strategy, the multifunctional and high-performance HMP papers have enormous potential for applications in aerospace, electromagnetic protection, and thermal management.

## Experimental Section

### Materials

Ti_3_AlC_2_ powder (400 mesh) was provided from 11 Technology Co., Ltd. (Jilin, China). Lithium chloride (LiCl, purity 99.9%), iron trichloride (FeCl_3_, purity 99%), lithium fluoride (LiF, purity 99.9%), and pyrrole monomer (Py, purity 99%) were all bought from Aladdin Reagent Co., Ltd. (Shanghai, China). N-N-dimethylacetamide (DMAc, purity 99.8%) and concentrated hydrochloric acid (HCl) were purchased from Sinopharm Co., China (Beijing, China). p-phenylenediamine (PDA, purity 99.99%) was supplied from Amino-Chem Co., Ltd. (Shanghai, China). 5-(6)-Amino-2-(4-aminobenzene)-benzimidazole (APBZ, purity 99.9%) was received by Changzhou Sunlight Medical Co., Ltd. (Changzhou, China). Terephthaloyl chloride (TPC, purity 99.99%) was obtained by Shandong Kaisheng New Materials Co., Ltd. (Zibo, China).

### Preparation of Ti_3_C_2_T_x_ MXene Nanosheets and MXene/DMAc Dispersion

Ti_3_C_2_T_x_ MXene nanosheets were produced by selective etching Ti_3_AlC_2_ powder in LiF/HCl mixture through modified minimally intensive layer delamination (MILD) technology. Firstly, LiF (3.2 g) was slowly dissolved in 9 M HCl (40 mL) in a Teflon beaker while stirring for 5 min. Then, Ti_3_AlC_2_ MAX phase power (2 g) was slowly added to the above etchant, and the reaction was carried out at 40 °C for 24 h to etch the Al layer of the MAX phase completely. Subsequently, the resulting suspension was washed repeatedly with deionized water and centrifuged 6-8 times at 3500 rpm for 5 min until the pH was close to 6. The obtained dispersion was sonicated in an ice bath for 1 h under argon protection and centrifuged at 3500 rpm for 1 h to collect the MXene aqueous solution. The MXene/DMAc dispersions were obtained using the solvent exchange method. The prepared MXene aqueous dispersions were centrifuged at 10,000 rpm for 1 h to collect the sediment and then dispersed into DMAc. Finally, the above process was repeated three times, and the MXene/DMAc dispersion was successfully prepared.

### Polymerization of Heterocyclic Aromatic (HA) Solution

PDA, APBZ, and TPC polycondensation were used to obtain the HA solution using the DMAc/LiCl complex solvent. First, LiCl (2.45 g) was added to DMAc (67.55 g) in a two-necked flask and stirred under nitrogen protection until LiCl was fully dissolved. Then, PDA (0.005 mol) and APBZ (0.005 mol) monomers were added to the above solvent system and stirred for 1 h until completely dissolved. Finally, TPC (0.01 mol) was added to the solution, and stirring was continued for 1 h at 0-5 °C and 2 h at 30 °C to acquire a light yellow viscous polymer solution.

### Preparation of HA/MXene@PPy (HMP) Papers

First, the HA solution was added to the MXene/DMAc dispersion with continuous stirring to form a uniform and stable sol. The black sol was poured into a clean mold; the excess sol was scraped off with a blade and then immediately immersed in an ethanol bath for 2 h to form a smooth HA/MXene gel. The gel was submerged in water to exchange solvent for 2 h, repeated six times to completely remove ethanol and DMAc and ultimately obtain HA/MXene hydrogel. Subsequently, the HA/MXene hydrogel was pre-soaked in Py solution with different concentrations and vibrated with a shaker under an ice-water bath at 200 rpm. FeCl_3_ was added to the above system after pre-soaking for 1 h and polymerized for 1.5 h to prepare HMP hydrogel. Then, the formed HMP hydrogel was pressed between two clean glass plates and vacuum dried at 50 °C for 24 h to acquire the HMP papers. The pristine HA, HA/MXene paper was prepared using a similar process for comparison. HA/MXene papers with different ratios (90/10, 80/20, 70/30, 60/40) were manufactured by adjusting the amount of HA and MXene, denoted as HM10, HM20, HM30, and HM40, respectively. HM30 was used as a mechanically robust template to prepare HMP nanocomposite paper due to its excellent mechanical properties in this work. Therefore, the HA and MXene content ratio in all HMP papers is 70/30. The PPy content in HMP papers was tunable with various pyrrole concentrations during polymerization. HMP papers with different mass fractions of PPy (8, 17, 25, 33, and 42 wt%) were prepared, marked as HMP8, HMP17, HMP25, HMP33, and HMP42, respectively. As a control sample, the HA@PPy paper with 33 wt% PPy loading (HP33) was also obtained using the same procedures for polymerizing PPy onto the HA template.

### Characterization

Detail is provided in the supporting materials.

## Results and Discussion

### Morphology and Structural Characterization of the HMP Nanocomposite Paper

**Fig. 1:**
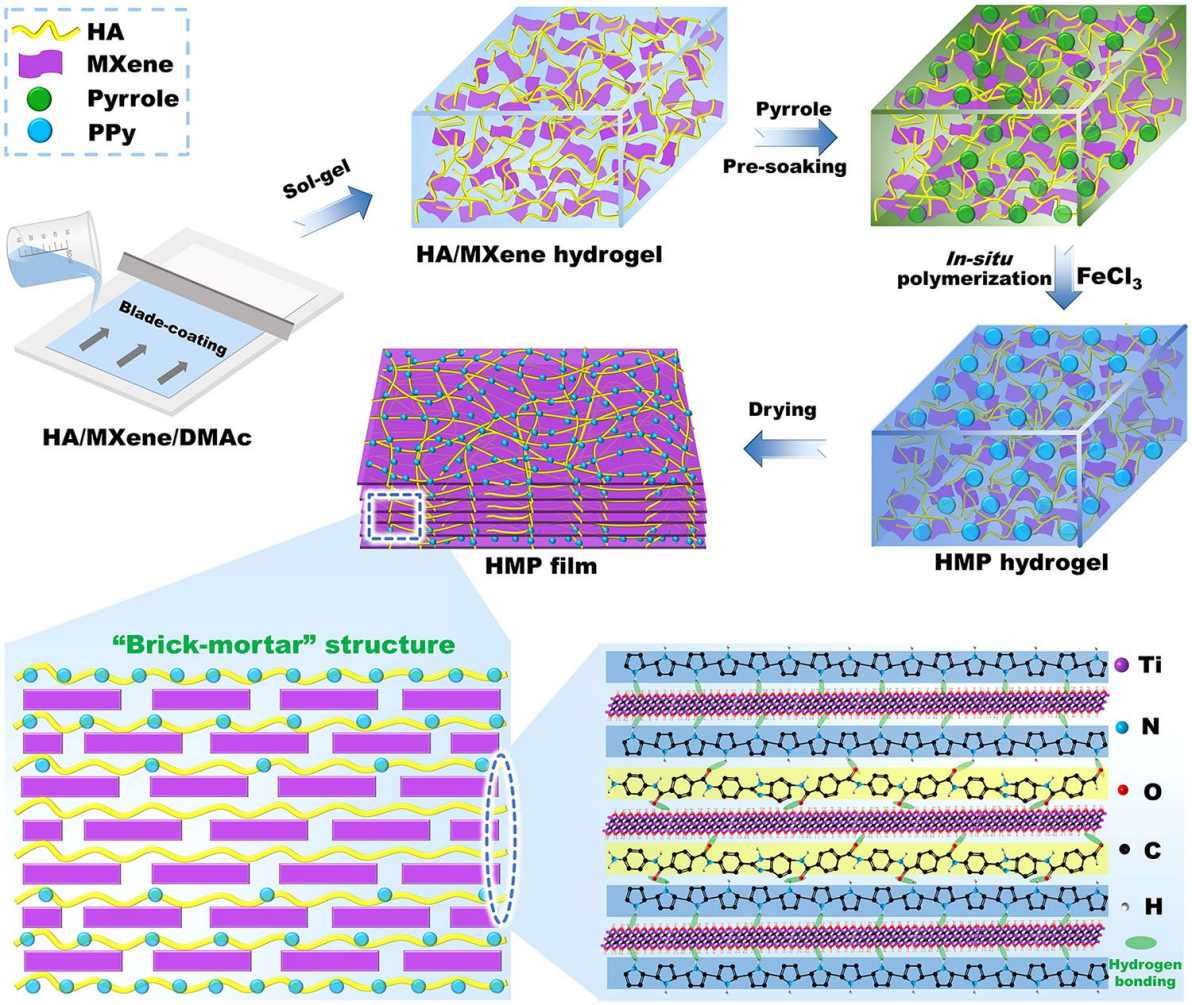
Preparation process and schematic diagram of HMP nanocomposite paper

**Fig. 2:**
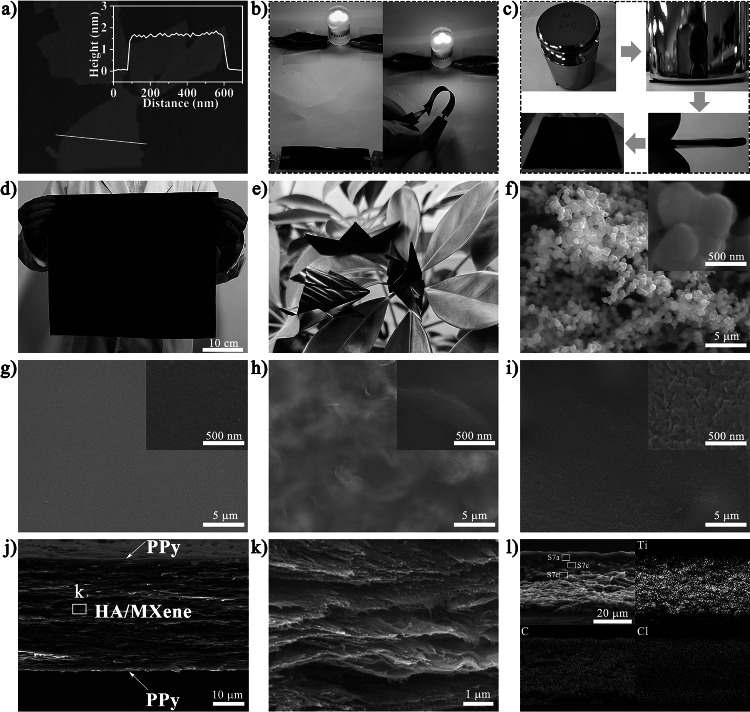
**a** AFM image and height profiles of MXene nanosheets. **b** Digital image of repeatedly bent conductive HMP hydrogel lighting up an LDE bulb. **c** Digital photographs of HMP hydrogel were folded in half and pressed without damage. **d** Optical photograph of large-scale HMP nanocomposite paper. **e** Flexibility diagram of HMP paper folded into "crane," "frog," and "ship" shapes. SEM images of **f** template-free polymerized PPy particles,** g** HA paper, **h** HA/MXene paper, and **i** HMP nanocomposite paper. **j, k** Cross-sectional SEM images of HMP nanocomposite paper. **l** Ti-, C-, and Cl- element mappings of the HMP paper cross section

The fabricating process for the HMP nanocomposite paper is schematically illustrated in Fig. 1. Firstly, Ti_3_C_2_T_x_ MXene flakes were synthesized by selective etching Ti_3_AlC_2_ MAX powder in LiF/HCl mixture followed by sonication; then, through high-speed centrifugation-organic solvent exchange technology, a highly stable MXene/DMAc dispersion was obtained (Fig. S1) [26]. Scanning electron microscopy (SEM) image of thoroughly exfoliated ultrathin MXene nanosheets presented an average lateral size of 463.2 nm (Fig. S2). Meanwhile, atomic force microscopy (AFM) images and height profile mapping (Fig. 2a) showed that the thickness of the Ti_3_AlC_2_ nanosheet was approximately 1.61 nm, which was equivalent to a single-layer MXene, suggesting that the MXene nanosheets were successfully exfoliated [27]. In addition, the polymer chains of HA were copolymerized production in the DMAc/LiCl complex solvent from three monomers: p-phenylenediamine (PDA), 5-(6)-Amino-2-(4-aminobenzene)-benzimidazole (APBZ) and p-phthaloyl chloride (TPC) (Fig. S3) [28]. According to previously reported literature, the mechanical properties of HA paper were optimal when the molar ratio of APBZ to diamine was 1:1, so the molar ratio of APBZ to diamine was set to 1:1 [29]. After the reaction, a uniform and stable light yellow HA solution was finally successfully synthesized. Surprisingly, the resulting MXene dispersions and HA solution exhibited a strong Tyndall effect, revealing good colloidal properties and dispersion (Fig. S4a, b). Subsequently, the HA solution was added to the MXene/DMAc dispersion with continuous stirring to form a uniform and stable sol, as demonstrated by the Tyndall effect (Fig. S4c). Pour the black sol into a clean mold and remove excess sol by scraping with a blade, then immediately immerse in an ethanol bath to form a smooth HA/MXene gel, serving as a template for the assembly of conducting polymers (Fig. S5a). After washing with deionized water to remove excess DMAc and ethanol, the HA/MXene hydrogel was pre-soaked in Py solution with different concentrations and vibrated with a shaker under an ice-water bath at 200 rpm. FeCl_3_ was added to the above system after pre-soaking for 1 h, then polymerized for 1.5 h to obtain HMP hydrogel (Fig. S5b). The unique penetration process of PPy gave HMP hydrogel high and stable conductivity, which could light up LED lights regardless of bending or unfolding at a low voltage (Fig. 2b). In addition, the folded HMP hydrogel could still bear a weight of 2 kg without breakage (Fig. 2c), displaying excellent flexibility. Large-sized free-standing HMP nanocomposite paper was successfully obtained after further drying in a vacuum (Fig. 2d). The prepared HMP papers could be folded into different shapes (Fig. 2e), demonstrating their superior flexibility and folding resistance.

As shown in Fig. 2f, PPy particles polymerized in aqueous media tend to be randomly distributed due to the absence of template assistance. The pure HA paper exhibited a smooth surface (Fig. 2g), while the HA/MXene paper surface presented a slightly wrinkled structure due to the introduction of MXene (Fig. 2h). On the other hand when there was HA/MXene template-assisted polymerization, a dense layer of PPy was observed to be evenly and regularly dispersed on the paper surface (Fig. 2i), suggesting that the template effect could effectively guide the *in-situ* assembly of PPy. Moreover, the cross-sectional SEM images of the HMP paper presented an apparent sandwich structure composed of HA/MXene in the middle and PPy on the surface (Fig. 2j). The HA/MXene layer exhibited a nacre-inspired "brick-and-mortar" lamellar structure (Fig. 2k), indicating that the MXene nanosheets interact closely with HA through hydrogen bonds. Besides, energy-dispersive spectrometer (EDS) mapping (Fig. 2l) of the HMP paper revealed that the Ti elements in the middle layer were evenly distributed, and almost no Ti elements were on the surface, implying that PPy was successfully assembled onto the HA/MXene template and the MXene was uniformly distributed in the template. The distribution of O elements also further confirmed this result (Fig. S6). At the same time, the Cl elements decreased in a gradient from the surface to the middle of the paper, indicating that the PPy was not only retained on the surface of HA/MXene but also penetrated the interior structure of the HA/MXene. High-definition cross-sectional SEM images of HMP nanocomposite paper further demonstrated these results. In the HMP paper, the PPy content on the surface is significantly higher than in the middle (Fig. S7). In addition, MXene, HA, and PPy are closely adhered to each other due to the strong hydrogen-bonding interaction between them, forming a "brick-and-mortar" structure with MXene as bricks and PPy and HA as mortar (Fig. S7e, f).

**Fig. 3:**
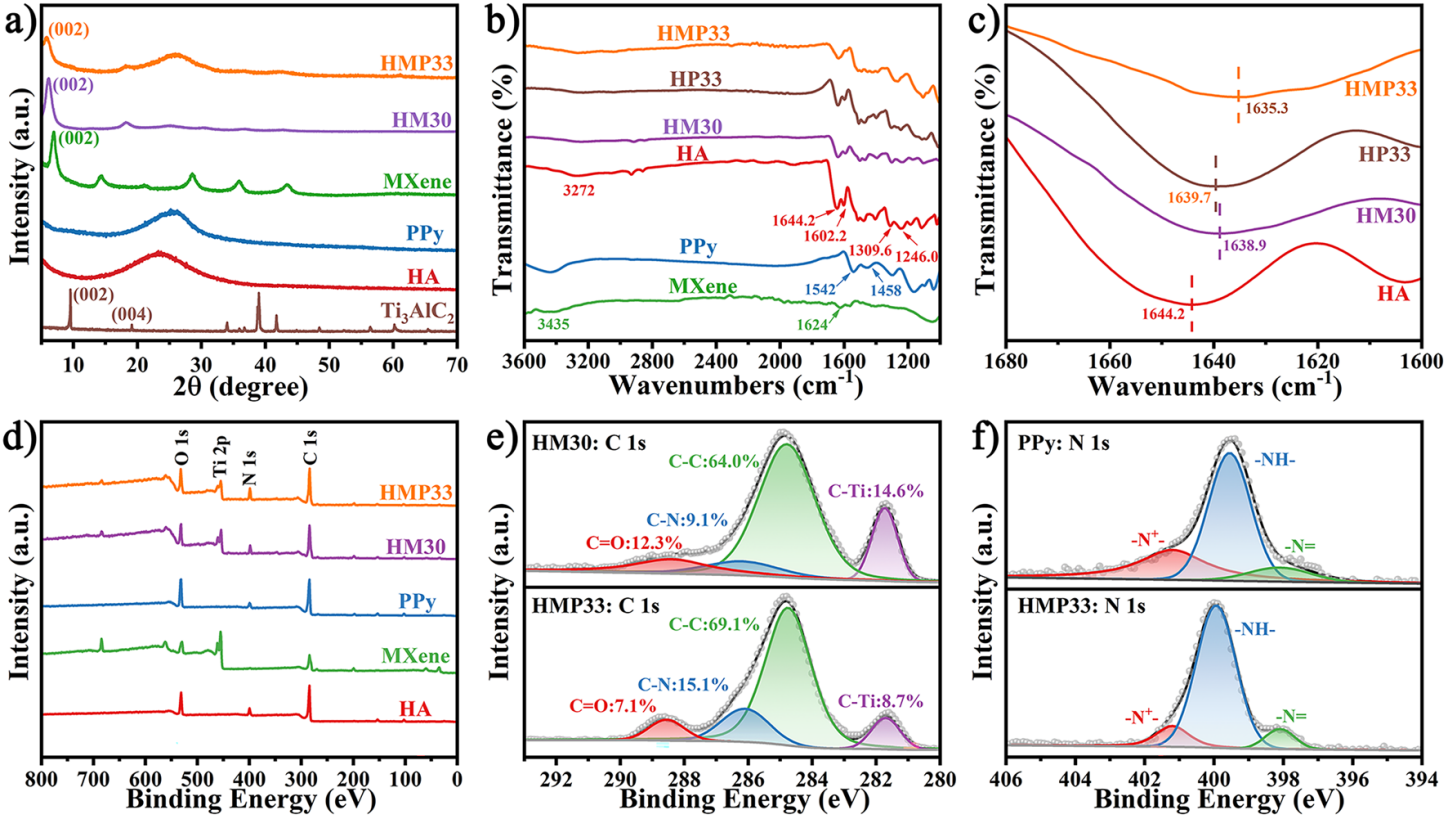
**a** XRD spectra, **b, c** FTIR spectra, and **d** XPS wide-scan spectra of various papers. High-resolution XPS spectra of **e** C 1*s* and **f** N 1*s*

The X-ray diffractometer (XRD) patterns of samples are displayed in Fig. 3a. A typical single broad diffraction peak of HA was detected at 23.6°, demonstrating that HA was successfully polymerized [30]. At the same time, the disappearance of the (104) peak and the increase in the lamella spacing caused by the movement of the (002) peak to a small-angle direction proved the complete etching of the Al layer and the successful delamination of the MXene nanosheets, respectively [31]. Compared with the pure MXene paper, the (002) characteristic peak of HA/MXene paper shifted from 6.9° to 6.1° due to the incorporation of HA, suggesting that HA was successfully introduced into the interlayer of MXene flakes. Furthermore, the presence of crystal planes of HA and PPy hybrids and the shift of the (002) diffraction peak from 6.1° to 5.8° in HMP demonstrated that PPy was not only successfully loaded on the surface of the HA/MXene but also penetrated the interior of the layered HA/MXene template. Notably, the change in lamellar spacing was convincing evidence for successfully embedding HA and PPy into the MXene layer. These results were further demonstrated by the FTIR spectra, as illustrated in Fig. 3b. The absorption bands of MXene at 1624 and 3435 cm^−1^ were attributed to the vibration absorption of C-O and -OH, respectively [12]. In addition, the characteristic peaks of HA at 3272 and 1644.2 cm^−1^, respectively, represented the stretching vibration of N-H and C = O in the amide groups. The absorption bands at 1246.0, 1309.6, and 1602.2 cm^−1^ represented the benzimidazole group's N-H, C-N, and C = N vibrations, suggesting the successful polymerization of the third monomer APBZ [32]. For HMP, absorption peaks that appeared in 1542 and 1458 cm^−1^ were attributed to C = C and = C-N symmetric and asymmetric ring stretching, respectively, revealing that PPy was successfully polymerized [33]. Through local amplification (Fig. 3c), the characteristic peaks of C = O in HA/MXene showed an obvious red shift, demonstrating the existence of strong hydrogen bond interaction between HA and MXene. Moreover, a larger distance redshift to 1635.3 cm^−1^ was observed in HMP nanocomposite paper, suggesting that PPy, HA, and MXene all had strong hydrogen bond interactions with each other.

The elemental composition and chemical structure of HMP and its components were further analyzed using X-ray photoelectron spectroscopy (XPS). Figures 3d and S8a results indicated that the surface and edges of MXene nanosheets contain abundant functional groups due to the introducing of terminal groups during the synthesis process. After incorporating HA and *in-situ* polymerization of PPy, the corresponding HMP XPS spectrum produced characteristic peaks of MXene, HA, and PPy simultaneously. As shown in Fig. S8b, the distinct peaks appearing at 533.5, 531.6, 530.5, and 529.7 eV of HMP nanocomposite paper were attributed to the C = O, C-Ti-(OH)_x_, C-Ti-O_x_, and Ti-O in the high-resolution O 1*s* spectra, respectively. The high-resolution XPS spectra of C 1*s* are presented in Fig. 3e, and the characteristic peaks at 288.6, 286.1, 284.8, and 281.7 eV were, respectively, assigned to the C = O, C-N, C-C, and C-Ti in the HMP nanocomposite paper. The increase in peak intensity at 286.1 eV also confirmed the presence of PPy [33]. For the high-resolution N 1*s* spectra (Fig. 3f), three distinct characteristic peaks at 401.2, 399.9, and 398.1 eV represented -N^+^-, -NH-, and -N = in the HMP composite paper, respectively. Besides, the binding energy of C-Ti-(OH)_x_ peak in O 1*s* at 532.1 eV of MXene shifted to 531.9 eV of HA/MXene. Meanwhile, the peak at 531.9 eV corresponding to C-Ti-(OH)_x_ further moved to 531.6 eV after loading PPy, indicating that the chemical environment of C-Ti-(OH)_x_ in HMP had changed [21]. More importantly, the characteristic peak at 288.4 eV in C 1*s* for the C = O group of HA/MXene moved to 288.6 eV for HMP, and the binding energy of the -NH- characteristic peak in N 1*s* shifted from 399.5 eV for PPy to 399.9 eV for HMP after the *in-situ* assembly of PPy. The shifts of C = O and -NH- in the direction of high binding energy suggest that PPy was not only successfully combined with HA/MXene but also formed a strong hydrogen bond effect between PPy and HA/MXene [34]. At the same time, the displacement of C-Ti-(OH)_x_ in the low binding energy direction confirmed that MXene, HA, and PPy had strong hydrogen bond interactions with each other, which helped to enhance the mechanical properties of the nanocomposite paper.

### Mechanical Properties and Fracture Behaviors of the HMP Nanocomposite Paper

**Fig. 4:**
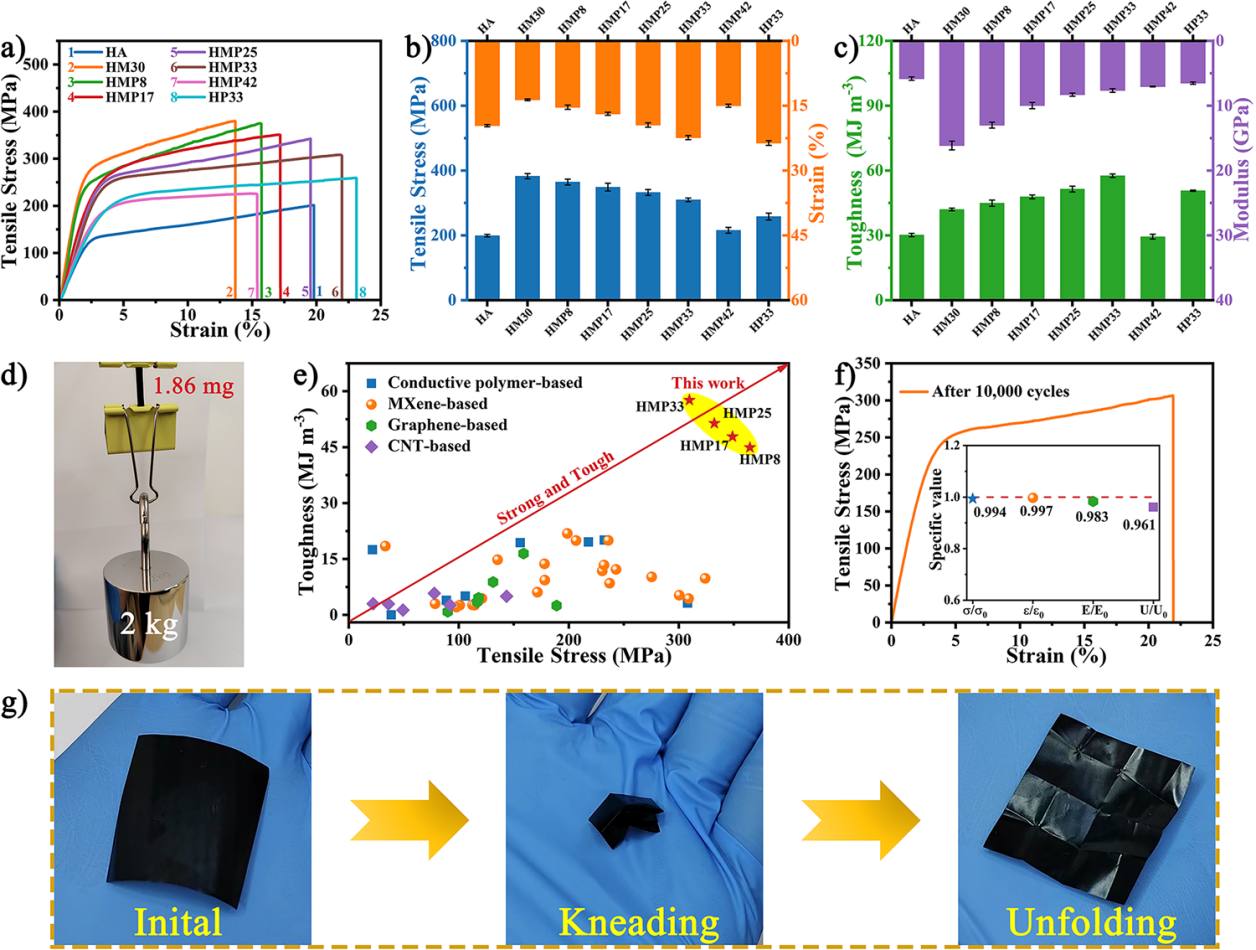
Mechanical properties and folding resistance of HMP nanocomposite papers. **a** Representative stress-strain curves and corresponding **b** tensile strength, strain, **c** toughness, and modulus of the HA, HM30, and HMP papers. **d** Digital photograph reveals the remarkable robustness of HMP paper. **e** Comparison of toughness and strength of nacre-like HMP paper with those of previously reported EMI papers (see Table S2 for details). **f** Tensile stress-strain curve of HMP33 paper after 10,000 bending-relaxation cycles. The tensile strength ratio (σ/σ_0_), elongation at break ratio (ε/ε_0_), toughness ratio (U/U_0_), and Young's modulus ratio (E/E_0_) are shown in the inset. **g** Digital photograph of the HMP33 paper kneaded by hands displaying no breakage

The outstanding mechanical properties of EMI shielding materials were of great significance, especially in flexible electronics, which require sufficient toughness to withstand various mechanical deformations. Typical stress–strain curves and the mechanical performances of various papers are presented in Figs. 4a-c, S9, and Table S1. Under uniaxial stretching, all papers exhibited similar nacre-mimetic tensile behavior, inducing linear elastic deformation first and then apparent significant plastic elongation until fracture (Figs. 4a and S9a). The pure HA paper displayed a tensile strength of 199.1 MPa, a strain of 19.6%, a fracture toughness of 30.2 MJ m^−3^, and a modulus of 5.8 GPa. With the introduction of MXene flakes, the HA/MXene papers substantially improved tensile strength and toughness (Fig. S9b, c). Especially when the MXene content was 30%, the strength and toughness of the HM30 composite paper reached the optimal values of 382.7 MPa and 42.0 MJ m^−3^, which were 192% and 139% higher than the pure HA paper, respectively. Therefore, layered HM30 was used as a mechanically robust template to prepare HMP nanocomposite paper with high strength and toughness. After the *in-situ* assembly of PPy, although the tensile strength and modulus of HMP papers were slightly reduced, their fracture strain and toughness were significantly increased. In particular, the HMP paper with 33 wt% PPy content (HMP33) achieved the optimal mechanical properties, including tensile strength, elongation at break, fracture toughness, and modulus of 309.7 MPa, 22.4%, 57.6 MJ m^−3^, and 7.6 GPa, respectively, which were 1.56, 1.14, 1.91, and 1.31 times those of the pure HA paper, respectively (Fig. 4b, c). In addition, the strength and toughness of the HMP33 paper were 120% and 114% higher than the HP33 paper, respectively, confirming the apparent superiority of the HA/MXene template compared to the HA template.

Besides, the excellent mechanical properties of the HMP nanocomposite paper were further proven by the fact that the lightweight paper will not break when lifting weight more than 1,000,000 times its weight (Fig. 4d). It was well known that strength and toughness were mutually exclusive for most composite materials. However, due to the optimized "brick-and-mortar" lamellar structure and strong hydrogen bond interactions between components, the HMP composite papers simultaneously exhibited splendid tensile strength and toughness compared with previously reported EMI shielding papers (Fig. 4e and Table S2). In addition, the HMP composite papers possessed outstanding fatigue resistance. Even after folding and unfolding 10,000 times, all mechanical property values of the HMP33 paper remained above 96% (Fig. 4f). More intuitively, it could also be seen that a piece of HMP33 paper was kneaded and rubbed by hands without any damage, further demonstrating the excellent folding durability (Fig. 4g and Movie S1). Besides, due to the *in-situ* assembly of hydrophobic PPy, the HMP paper obtained a higher water contact angle of 95.2° compared with MXene, HA, and HM30 papers (Fig. S10). Such excellent hydrophobicity and mechanical properties enabled the nanocomposite paper to have excellent anti-ultrasonic structural stability (Fig. S11), suggesting it could withstand harsh environments.

**Fig. 5:**
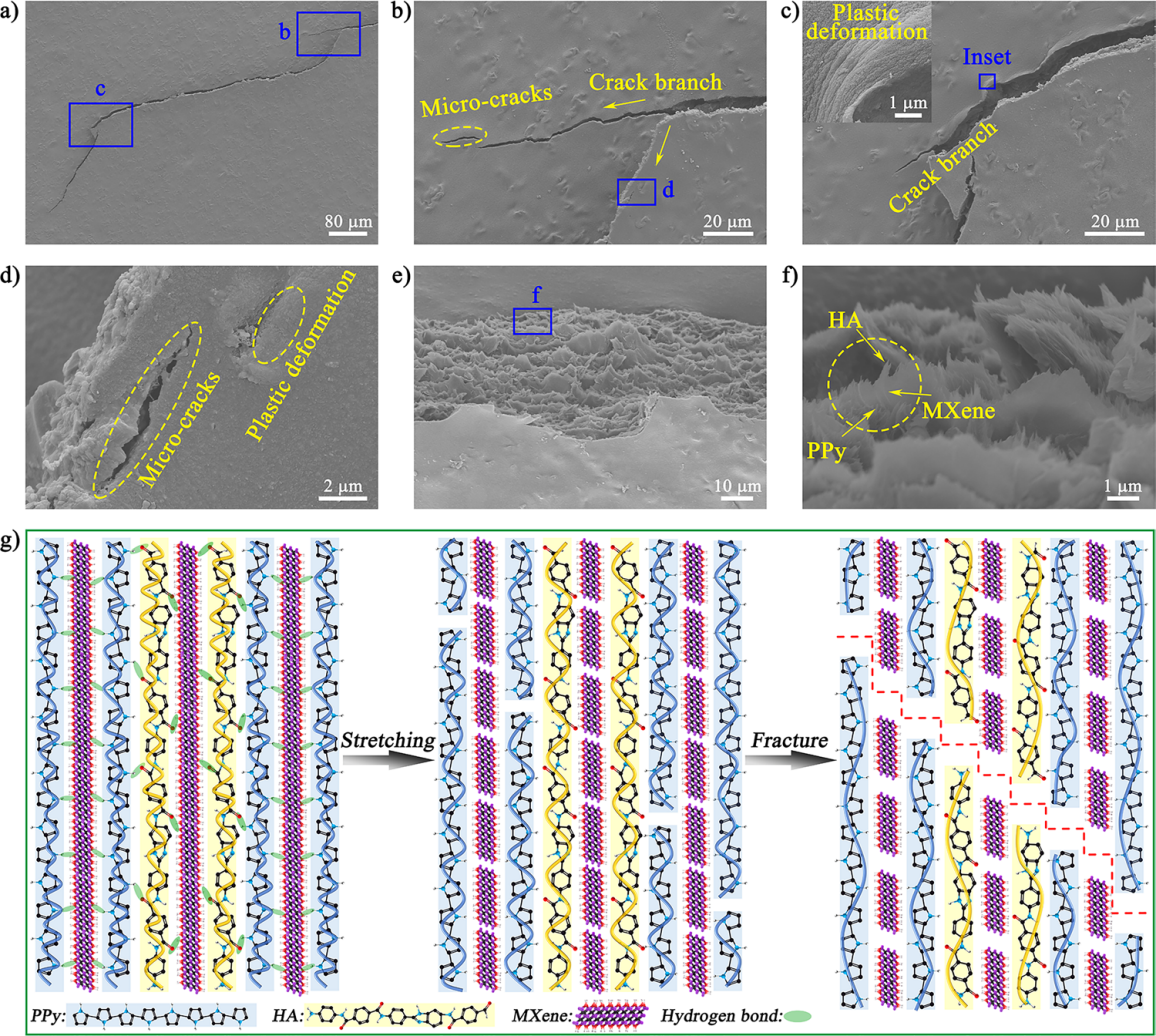
Fracture behaviors of the HMP papers. SEM images of **a-d** the crack propagation and **e, f** tensile fracture surface morphologies for HMP paper. **g** Schematic diagram of the crack propagation mode of the HMP papers

The fracture morphology and the crack propagation of HMP paper had been observed to reveal the strengthening and toughening mechanisms. The cracks in the HMP paper initiated from the notch and presented a zigzag-shaped expansion path (Fig. 5a). This representative long-range zigzag crack propagation phenomenon was regarded as one of the most critical external toughening mechanisms of nacre-like "brick-and-mortar" structural materials with high fracture resistance [35, 36]. *In-situ* assembly of polypyrrole provided more bridges between MXene nanosheets and HA polymer chain layers. During the stretching process, hydrogen bonds between components could also transfer stress to each other between layers. This fracture morphology provided direct and convincing evidence for a nacre-like lamellar design method to toughen HMP nanocomposite papers. The high-magnification SEM images showed that the nacre-like layered HMP paper also caused crack bifurcation, plastic deformation, and multiple cracks during the crack propagation process (Fig. 5b-d). Such abundant microscopic deformation could effectively retard the expansion of cracks and consume a large amount of energy, thereby significantly alleviating local excessive stress concentration [37, 38]. In addition, the crack path on the fracture surface showed a multi-level wave shape (Fig. 5e), further suggesting that the PPy and MXene slip under external stress enhanced the plastic elongation. The magnified view (Fig. 5f) revealed that many HA chains protruded from the edge of PPy and MXene along the stretching direction. This phenomenon further implied that the large deformation of the interconnected HA three-dimensional network was strongly associated with the sliding of MXene and PPy, achieving efficient energy consumption via the friction effect, thereby resisting the damage of tensile stress [39]. In contrast, the crack propagation of pristine HA paper showed linear extension (Fig. S12). The "brick-and-mortar" structure design and interface interaction played an essential role in enhancing nacre-like paper's energy consumption and fracture toughness.

The crack propagation mode and fracture mechanism of the nacre-inspired structure paper with significant elongation at break and ultrahigh toughness are more intuitively shown in Fig. 5g. In the early stages of stretching, the hydrogen bondings among the components of the HMP paper were first destroyed, such as the hydrogen bondings between C = O on HA and N-H on PPy, hydrogen bondings between C = O on HA and -OH on MXene, hydrogen bondings between -OH on MXene and N-H on PPy, resulting in interface slippage among PPy, MXene, and HA [40]. The curved HA in the frame straightened and released its hidden length. In this process, the HA framework distributed stress uniformly and maintained the macroscopic sample's integrity due to HA's high interconnectivity. As the external stress further increased, the friction effects of HA, MXene, and PPy limited the sliding of MXene and PPy and the formation of microcracks in the HMP paper, consuming a large amount of energy [4, 41]. When the hidden length of bent HA was exhausted, the HA framework was eventually broken, and HA, MXene, and PPy were pulled out together.

### Electrical and EMI Shielding Properties of the HMP Nanocomposite Paper

**Fig. 6:**
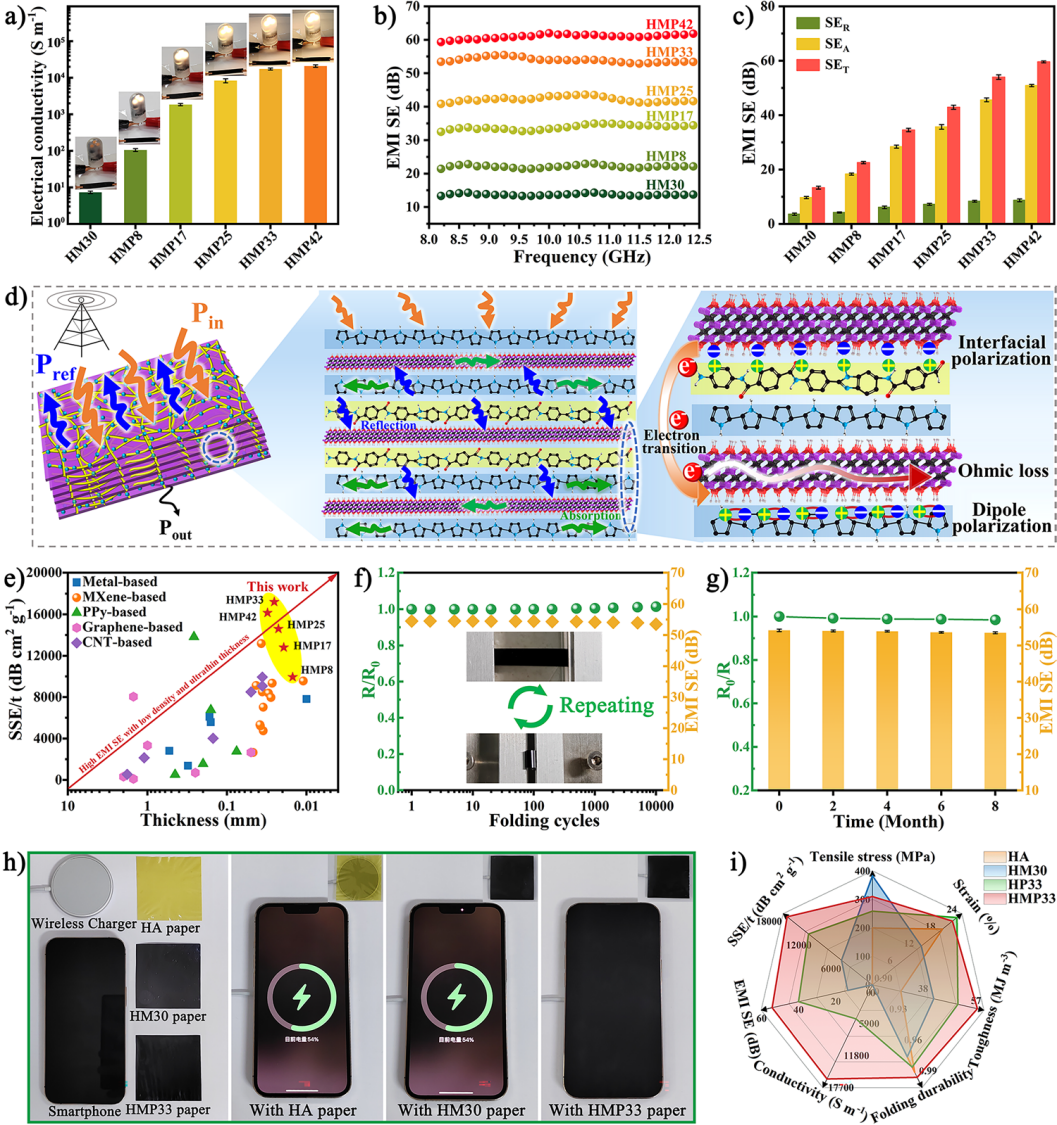
Electrical conductivity and EMI shielding performance of the HMP papers. **a** Electrical conductivity of the HM30 and HMP papers with different loading. **b** EMI SE values of the HMP papers with PPy contents of 0, 8, 17, 25, 33, and 42 wt%, respectively, correspond to thicknesses of 13.3, 14.9, 19.3, 22.5, 25.4, and 30.9 µm. **c** Comparison of SE_T_, SE_A_, and SE_R_ values of the HMP papers. **d** EMI shielding mechanism diagram of HMP papers. **e** SSE/t versus thickness of the HMP papers compared with previously published EMI shielding materials. Resistance changes and EMI SE of the HMP33 paper after **f** folding-unfolding cycles and **g** after being placed for 8 months without vacuum protection. **h** Digital images present the EMI shielding effect of various papers between smartphones and wireless chargers. **i** A radar chart presents performance comparisons from various papers

As we all know, the EMI shielding performances of composite material were closely related to its electrical conductivity. HA paper was an insulating material with an ultra-low electrical conductivity of 8.2 × 10^–11^ S m^−1^ (Table S3). With the addition of MXene nanosheets, the HM30 paper exhibited a significantly improved electrical conductivity of 7.3 S m^−1^, changing from an insulator to a conductor. More importantly, after *in-situ* loading PPy, the electrical conductivity of the HMP nanocomposite papers increased rapidly with increasing PPy content (Fig. 6a). The HMP paper's electrical conductivity improved at a PPy content of 8, 17, 25, 33, and 42 wt% to 105.6, 1848.8, 8355.7, 17,386.2, and 20,914.0 S m^−1^, respectively, which far exceeds the conductivity required (1.0 S m^−1^) for EMI shielding materials in practical applications [14]. There were two major reasons: (i) The template effect of the layered structure guided the assembly of conductive polymers, which was expected to achieve an independent conductive PPy layer. At the same time, introducing MXene could prevent HA's inherent insulation from affecting PPy conductivity; (ii) the higher the PPy load in the HMP nanocomposite paper, the more effective conductive path could be formed, thereby obtaining a more considerable electrical conductivity value. The change in brightness of the light-emitting diode (LED) lamp further confirmed the change in electrical conductivity of the HMP nanocomposite paper (inset in Fig. 6a).

From the EMI SE test of the samples in the X-band (8.2-12.4 GHz), the pure HA paper showed no EMI shielding performance, which was significantly improved by the introduction of MXene nanosheets (Fig. 6b and Table S3). Moreover, the EMI SE values of HMP were remarkably higher than those of HM30, demonstrating that a load of PPy effectively enhanced the shielding ability of HM30. Meanwhile, the EMI SE improved from 13.3 to 59.6 dB with increasing PPy content, ascribed to enhanced electrical conductivity at higher PPy contents. Thus, EMI SE could be extensively controlled by effortlessly adjusting the PPy loading. In addition, the EMI SE values of all HMP papers exceeded 20 dB, and the shielding efficiencies were over 99.45% in the X-band (Fig. S13), meeting the requirements for commercial EMI shielding applications [42]. The calculated total EMI SE (SE_T_), reflected EMI SE (SE_R_), and absorbed EMI SE (SE_A_) values based on the measured *S*-parameters are presented in Fig. 6c. Both SE_R_ and SE_A_ showed a clear upward trend with the growth of PPy content. The improvement of SE_R_ was attributed to the further enhancement of electrical conductivity, whereas that of SE_A_ might be thanks to the increase in ohmic loss and polarization loss [43, 44]. In addition, the R values of all samples were better than the A values, suggesting that reflection loss dominated the shielding mechanism of HMP nanocomposite papers (Fig. S14).

The enhancement mechanism of the HMP composite was explored by measuring the complex magnetic permeabilities (Fig. S15) and permittivities (Fig. S16). Magnetic loss (tan *δμ*  =*μ"*/*μ'*) and dielectric loss (tan*δε * = *ε"*/*ε'*) reflect the magnetic loss and dielectric loss capabilities of the material, respectively [45]. After in-situ loading PPy, the tan*δ*_*μ*_ of the HMP composite barely changed, but the tan*δ*_*ε*_ increased significantly. The enhancement of dielectric loss revealed that the HMP composite can effectively improve the dissipation of electromagnetic waves (EMWs). As displayed in Fig. 6d, an illustration of the EMI shielding mechanism of HMP papers was provided. The remarkable EMI shielding properties of HMP papers were attributed to their highly conductive pathways and unique "brick-and-mortar" lamellar structure. When EMWs were incident on the surface of the HMP paper, most EMWs were immediately reflected due to the impedance mismatch between the HMP paper and air. Then, the highly connected conductive network constructed by PPy and MXene provided high electron density to generate current, causing ohmic loss and absorbing another part of EMWs [46]. Meanwhile, dipole polarization and interface polarization were also beneficial to the absorption of EMWs [47]. Moreover, the multi-layer structure helped EMWs to reflect or scatter back and forth between the layers, causing the EMWs' attenuation phenomenon to occur repeatedly, thus increasing the dissipation of EMWs. At last, the EMWs were effectively shielded due to the close cooperation of reflection, absorption, and multiple internal scattering or reflections, giving HMP papers excellent EMI shielding performance.

Density and thickness were essential for EMI shielding materials in aerospace, portable, and electronic device miniaturization applications [45]. Therefore, specific EMI SE (SSE/t, SE divided by the sample thickness and density) was usually used as a standard to evaluate EMI shielding ability, thereby avoiding the influence of density and thickness in different systems [8]. As shown in Fig. S17, the SSE/t values of HMP paper significantly increased with increasing PPy loading and obtained an optimal value of 17,204.7 dB cm^2^ g^−1^ at a PPy loading of 33 wt%, surpassing the performance of most previously published EMI shielding materials (Fig. 6e and Table S4). Meanwhile, the SSE/t value of the HMP paper was significantly better than the HM30 and HP33 paper, indicating that its excellent EMI performance resulted from the synergistic effect of MXene and PPy. Furthermore, from the perspective of practical applications, composite papers are needed to have stable and reliable EMI shielding properties in various complex environments. As shown in Fig. 6f, the relative resistance (R/R_0_) and EMI SE of HMP paper remained at about 98.6% and 98.2% after 10,000 bending-relaxation cycles, respectively, demonstrating the effect of strong hydrogen bonds among components and "brick-and-mortar" structure gave the HMP paper with outstanding electrical stability and splendid fatigue resistance. Besides, even after aging for 8 months, the resistance of the paper had almost no change. Its EMI SE value only decreased by 1.3%, suggesting that the HMP paper possessed remarkable aging resistance and oxidation resistance (Fig. 6g). This result was mainly attributed to the dense and hydrophobic PPy layer preventing the penetration of O_2_ and H_2_O. After being soaked in deionized water, NaCl solution, and strong acid for 7 days, the material maintained an outstanding SE value (Fig. S18), further proving its durability and stability under harsh conditions. To verify the EMI shielding ability of the HMP paper under actual conditions, the power transfer during wireless charging of a smartphone was studied. The charging effect was evaluated by placing HA, HM30, and HMP33 papers of the same thickness between the smartphone and the wireless charger (Fig. 6h). The smartphone showed normal charging status under the influence of HA and HM30 papers but failed to charge when covered by HMP33 paper, proving that HMP paper had an excellent shielding effect on external EMW radiation. Figure 6i shows the radar chart for performance comparison, which proved the rationality of the "brick-and-mortar" microstructure construction of the HMP paper and the layered structure template to guide the assembly of conductive polymers, demonstrating the superiority of macroscopic properties, including outstanding mechanical properties, high durability, and high-performance EMI shielding.

### Joule Heating Performance of the HMP Nanocomposite Paper

**Fig. 7:**
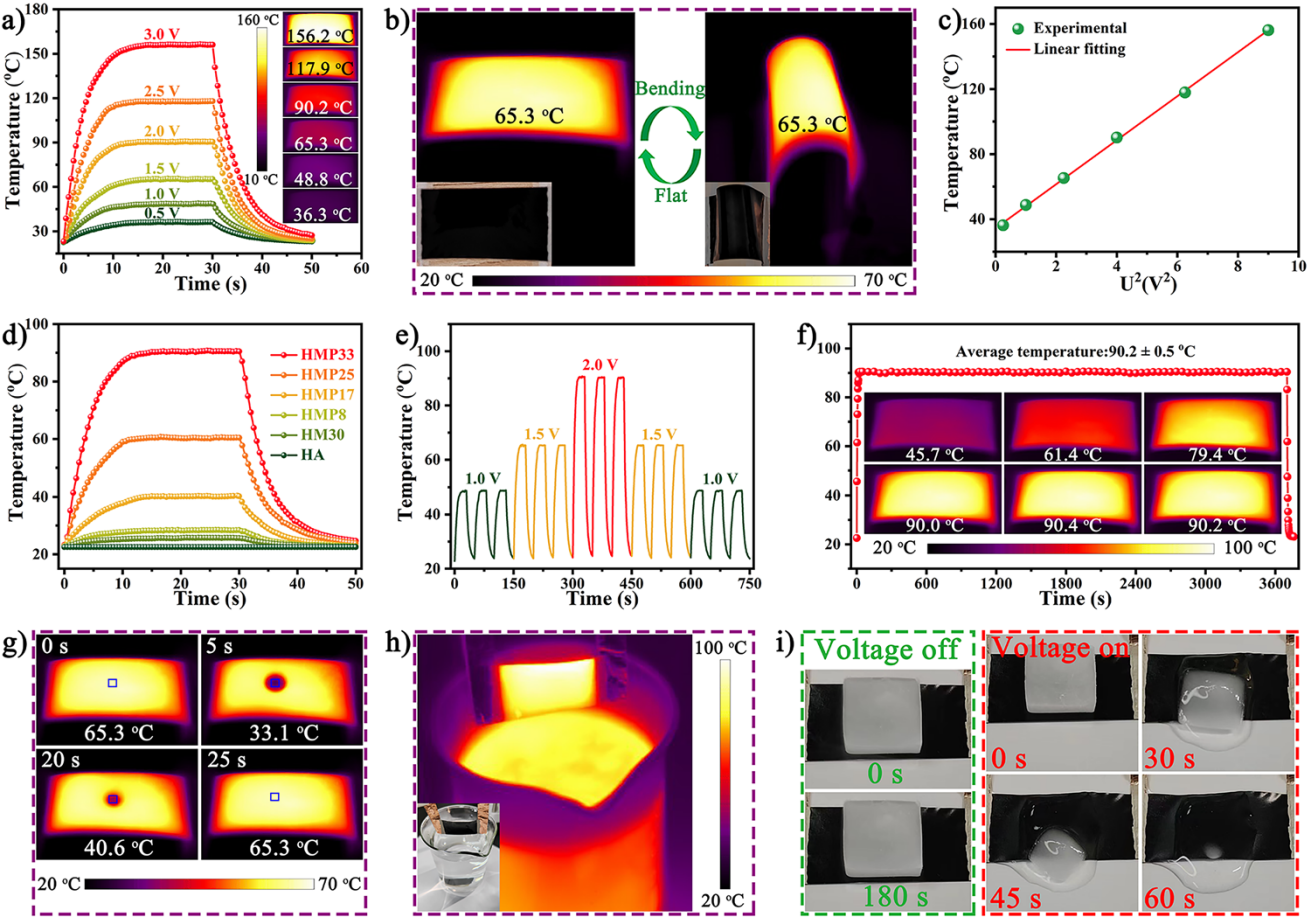
Electrothermal performance of the HMP papers. **a** Electrical heating performance of HMP paper under different driving voltages (insets correspond to IR thermal images). **b** IR thermal images of unfolded and bent paper heater (insets correspond to digital images). **c** The curve of surface temperature to *U*^*2*^. **d** Surface temperature curves of the HMP papers with various PPy contents under 2.0 V voltage. **e** Cyclic responsiveness of the HMP33 paper under 1.0, 1.5, and 2.0 V. **f** Temperature stability of the HMP33 paper at 2.0 V. The IR thermal image of HMP33 paper heaters upon **g** water drop and **h** steam treatments. **i** HMP paper de-icing process with voltage on/off

HMP nanocomposite paper with excellent electrical conductivity could also be a high-performance Joule heater, showing great potential in flexible wearable electronics [48]. An infrared (IR) camera was used to record the changes in the equilibrium temperature of HMP33 paper with time under low power supply voltage (Fig. 7a). The surface temperature quickly rose from room temperature to 36.3, 48.8, 65.3, 90.2, 117.9, and 156.2 °C at low voltages of 0.5, 1.0, 1.5, 2.0, 2.5, and 3.0 V, respectively. At the same time, the IR thermal image in the illustration revealed a uniform temperature distribution, which was regarded as a critical indicator of excellent electric heaters. Even more satisfying was that the flexible and wearable HMP paper still had a uniform temperature distribution under repeated bending (Fig. 7b). As shown in Fig. 7c, the equilibrium temperature and the square of the supply voltage (*U*^*2*^) of the HMP33 paper revealed a satisfactory linear relationship consistent with Joule's law [48], indicating a controllable electrothermal property. Furthermore, *in-situ* loading of PPy could significantly improve the electrothermal performance of HM30 paper. The equilibrium temperature of HMP paper gradually increased with increasing PPy content due to improved electrical conductivity (Fig. 7d). The saturation temperature of the HMP paper exhibited regular and reversible changes through cyclic application of different voltages, indicating that its electrothermal performance had outstanding cycle stability (Fig. 7e). The temperature of the HMP paper heater rapidly rose to the saturation temperature of about 90.2 °C and remained steady even after 1 h, demonstrating high responsiveness and durable stability (Fig. 7f). Figure 7g shows water droplets used to simulate raindrops evaporating quickly on the HMP paper heater at a low voltage. Due to the excellent hydrophobicity and electrothermal properties of HMP33 paper, water droplets were heated and evaporated, proving the electrothermal stability in rainy environments. In addition, the stability and durability of the HMP paper heater under high-temperature and high-humidity environments were further confirmed by the stable and consistent Joule heating characteristics that could be obtained even if the HMP paper was placed in steam (Fig. 7h). Meanwhile, the melting of an ice cube placed on the HMP paper at a low voltage was observed to verify the feasibility of the HMP heater as a de-icing device in practical applications. The ice cube could melt and slide within 60 s thanks to the remarkable electrothermal performance and the low friction of the paper's hydrophobic surface (Fig. 7i). In short, even when used under extremely harsh conditions of high temperature, high humidity, low temperature, and freezing, the HMP paper heater still demonstrated superiority and excellent Joule heating performance.

### Photothermal Performance and Flame Resistance of the HMP Nanocomposite Paper

**Fig. 8:**
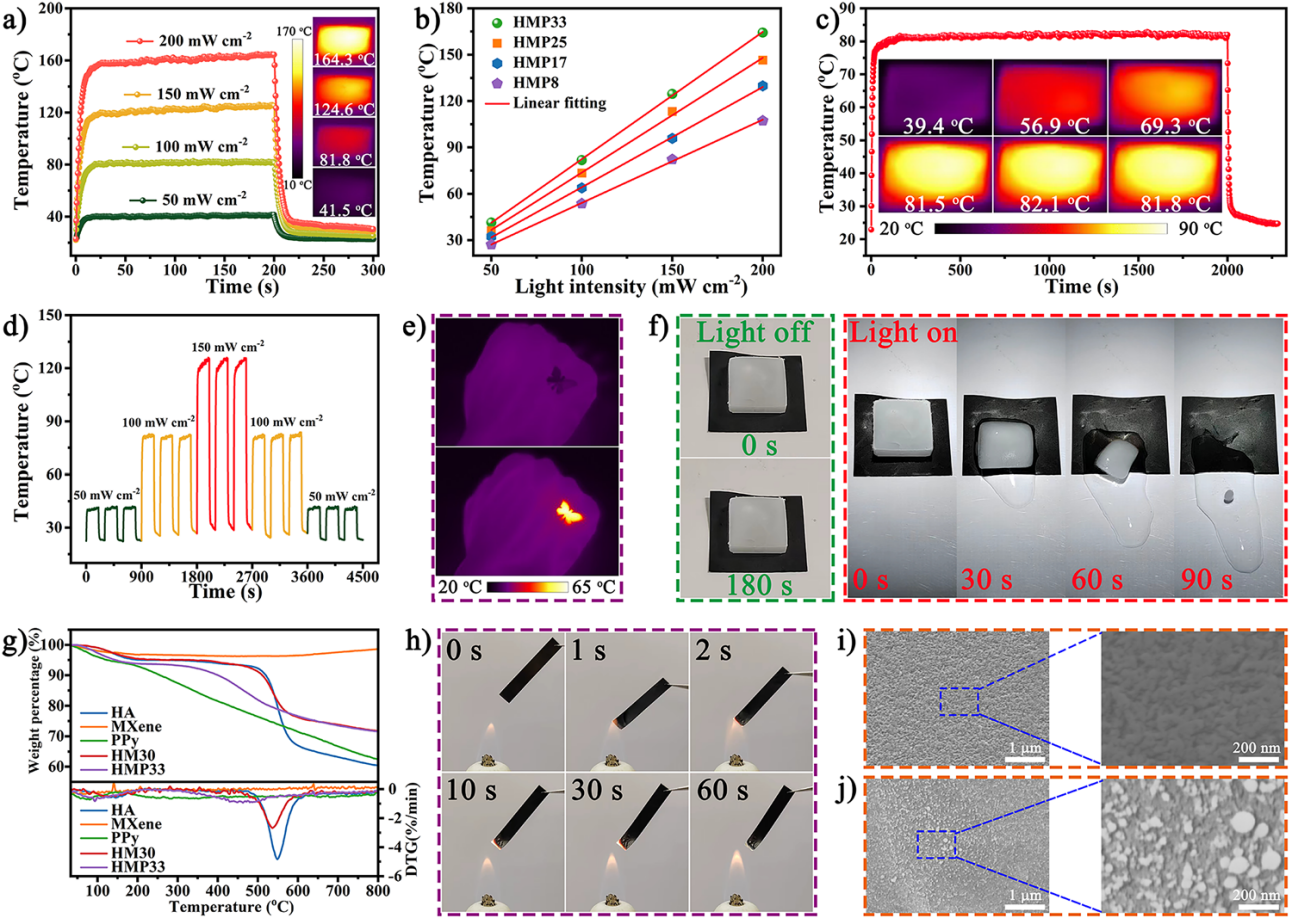
Photothermal properties, thermal stability, and fire safety of HMP papers. **a** Surface temperature curves of the HMP paper at various light intensities. **b** Relationship between equilibrium temperature and light intensity of HMP paper with different PPy contents. **c** Temperature stability of HMP paper under one sun illumination for 2000 s (insets correspond to IR thermal images). **d** Cyclic responsiveness of the HMP33 paper at 50, 100, and 150 mW cm^−2^. **e** IR thermal images of butterfly-shaped paper used as wearable heat therapy. **f** The HMP paper de-icing process with the light on/off. **g** TGA and DTG curves of PPy, HA, MXene, HM30, and HMP33 papers. **h** Optical photograph of HMP33 paper ablated under an alcohol lamp displays fire resistance performance. SEM images of the HMP33 paper **i** before and **j** after burning

PPy and MXene had the fascinating advantages of strong solar light absorption and efficient photothermal conversion, which enabled composites based on PPy and MXene to convert and utilize inexhaustible solar energy directly [49, 50]. In addition, the unique nacre-mimetic lamellar structure of the HMP paper contributed to multiple absorption, reflection, and scattering of the incident sunlight, enhancing its photothermal conversion capacity [6]. Under xenon lamp-simulated sunlight, the higher surface temperature of HM30 paper compared to pure HA paper was attributed to the excellent photothermal performance of MXene. More importantly, the equilibrium temperature of the HMP paper showed a sharp increase trend after loading PPy (Fig. S19). When the PPy loading increased from 8 to 33 wt%, the saturation temperature rose from 53.7 to 81.8 °C under one sunlight intensity. The saturation temperature had a linear relationship with the PPy loading (Fig. S20), indicating that PPy improved the sunlight absorption ability and played a leading role in the photothermal conversion process. In addition, when the simulated solar illumination intensity increased from 50 to 100, 150, and 200 mW cm^−2^, the saturation temperature of HMP33 paper rose almost linearly from 41.4 to 81.8, 124.6, and 164.3 °C, respectively (Fig. 8a). Therefore, the surface temperature of HMP paper could be easily adjusted by changing the PPy content or light intensity (Fig. 8b). As shown in Fig. 8c, the saturation temperature of HMP paper remained stable under continuous illumination for 2000 s at a light intensity of 100 mW cm^−2^, demonstrating long-term stability in photothermal performance.

Furthermore, when the xenon lamp with different light intensities was turned on or off, the temperature changes of the HMP paper were regular and reversible, indicating outstanding photothermal responsiveness, durability, and stability (Fig. 8d). Under IR thermal imaging, the surface temperature distribution of the HMP paper cut into a butterfly shape was uniform, indicating that intelligent heaters with reliable photothermal performance were easy to process and design (Fig. 8e). When the butterfly-shaped flexible paper adhered to human skin, a photothermal treatment temperature of 63 °C could be achieved at low light intensity. In addition, the photothermal therapy temperature could be easily adjusted by changing the light intensity of the flexible HMP heater. The excellent hydrophobicity and photothermal conversion ability also gave the HMP paper efficient de-icing capabilities. As shown in Fig. 8f, the ice cube placed on the paper did not melt within 180 s without lighting, while the ice cube could melt and slide within 90 s under illumination. In short, steady, controllable, high-performance photothermal conversion properties were achieved, which helps explore the multifunctional applications of HMP paper.

Thermal stability and fire safety were significant for developing high-performance EMI shielding materials that meet practical applications, especially under high-temperature conditions. The thermal stability of the HMP nanocomposite paper and each component was verified by the TGA and DTG analysis (Fig. 8g). The residual weight of the MXene was maintained above 98% at 800 °C, and there was no apparent maximum weight loss temperature, suggesting its excellent thermotolerance. Pure HA paper had only slight weight loss before 518 °C, owing to its inherent excellent thermal stability. After adding MXene, the residual weight of HM30 paper increased significantly, and the initial decomposition temperature reached 508 °C. In contrast, pure PPy exhibited an initial weight loss at approximately 140 °C due to the degradation of the polymer chain. After the *in-situ* loading of PPy onto the HA/MXene, the initial degradation temperature increased from 140 to 384.3 °C, while the residual mass was close to HM30, indicating that HMP paper possessed outstanding thermal stability. In addition, the fire safety of HMP paper was evaluated by alcohol lamp combustion experiments. After HMP33 paper was burned in a flame for 60 s, it only produced a small amount of residue and self-extinguished after leaving the fire source, showing remarkable flame retardancy (Fig. 8h). Subsequently, the surface micromorphology of the HMP paper before and after burning was observed through SEM images (Fig. 8i, j). Although a small amount of particles were produced on the surface of the burned HMP33 paper, it still maintained good structural integrity. These results might be due to two reasons: (i) The outermost PPy layer acted as a protective layer to isolate part of the oxygen and inhibit the fragmentation of HA/MXene; (ii) the oxidative decomposition of MXenes formed titanium dioxide and amorphous carbon during the combustion process, creating a barrier layer that effectively inhibited the transfer of heat and flame. In short, the outstanding thermal stability and flame resistance of HMP paper exceptionally ensured its safety and reliability in actual use, especially in high-temperature environments.

## Conclusions

In summary, a large-scale, durable, and multifunctional nacre-like HMP nanocomposite paper was fabricated via HA/MXene template-assisted *in-situ* assembly of PPy. The "brick-and-mortar" lamellar structure and the strong hydrogen bonds effect among MXene, HA, and PPy gave the paper remarkable mechanical properties, including high tensile strength, large strain, splendid fracture toughness, foldability, and anti-ultrasound. At the same time, HA/MXene templates were also demonstrated to efficiently assemble conductive polymer nanostructures to construct highly conductive nanocomposite papers. Thus, the HMP paper achieved high electrical conductivity, superior SSE/t of approximately 17,204.7 dB cm^2^ g^−1^, and excellent electrothermal performance (up to 156.2 °C at 3 V). Furthermore, the paper also exhibited outstanding photothermal performance (up to 81.8 °C at 100 mW cm^−2^), thermal stability, and fire resistance. These provided a simple and scalable strategy to design and prepare multifunctional nanocomposite papers with broad application prospects in electromagnetic protection, electrothermal/photothermal de-icing, thermal physiotherapy, and fire safety.

## Supplementary Information

Below is the link to the electronic supplementary material.

Supplementary file1 (DOCX 3755 KB)

Supplementary file2 (MP4 10155 KB)
