# Supplementary material for: Multifunctional Nacre-Like Nanocomposite Papers for Electromagnetic Interference Shielding via Heterocyclic Aramid/MXene Template-Assisted In-Situ Polypyrrole Assembly
Source: Nanomicro Lett. 2024 Oct 31;17:53. doi: 10.1007/s40820-024-01552-9 (PMC11528091; doi:10.1007/s40820-024-01552-9)
Supplement: Supplementary file 1 — Supplementary file1 (DOCX 3755 KB) [file 40820_2024_1552_MOESM1_ESM.docx]

Supporting Information for

**Multifunctional Nacre-Like Nanocomposite Papers for Electromagnetic Interference Shielding via Heterocyclic Aramid/MXene Template-Assisted *In-Situ* Polypyrrole Assembly**

Jinhua Xiong^1^, Xu Zhao^1,^*, Zonglin Liu^1^, He Chen^1^, Qian Yan^1^, Huanxin Lian^1^, Yunxiang Chen^1^, Qingyu Peng^1,^*, Xiaodong He^1^

^1^ National Key Laboratory of Science and Technology on Advanced Composites in Special Environments, Center for Composite Materials and Structures, Harbin Institute of Technology, Harbin 150080, P. R. China

*Corresponding authors. E-mail: [zhaoxu1@hit.edu.cn](mailto:zhaoxu1@hit.edu.cn) (Xu Zhao); [pengqingyu@hit.edu.cn](mailto:pengqingyu@hit.edu.cn) (Qingyu Peng)

**S1 Characterization**

The morphologies and microstructures of HA, MXene, PPy, HA/MXene, and the HMP papers were characterized by scanning electron microscopy (SEM, GeminiSEM 500, Carl Zeiss, Germany). Atomic force microscopy (AFM, Bruker, Germany) image of Ti_3_C_2_T_x_ MXene nanosheets was recorded using a Bruker Dimension Icon. Fourier infrared (FTIR) spectra were measured by a Nicolet iS20 step scan instrument (Thermo Scientific, USA). The papers' X-ray photoelectron spectroscopy (XPS) spectra were analyzed on K-Alpha equipment (Thermo Scientific, USA). The water contact angle of the sample was performed using an optical contact angle meter (OCA20, Dataphysics, Germany). TGA testing was carried out on a simultaneous thermal analyzer (TG 209 F3 Tarsus, Netzsch, Germany) with a heating rate of 10 ^o^C min^-1^ in an N_2_ atmosphere. The paper's electrical resistance (R) was obtained with a Keithley 2700 sourcemeter (Cleveland, OH, USA) using the four-probe technique at room temperature to calculate the conductivity.

The prepared paper was cut into strips (20 × 5 mm^2^) with a blade to obtain the tensile test sample. A universal testing machine (5944, Instron, USA) was used for the tensile test of the paper, and the strain rate set at room temperature was 1.0 mm min^-1^. The mechanical properties of all samples were averaged from at least three test results. Δ*L* is the length change, and *L* is the initial gauge length, so the fracture strain is Δ*L*/*L*. Toughness can be calculated according to the following formula:

$$U=\int_{0}^{\varepsilon_{max}} \delta d\varepsilon(S1)$$

where δ represents stress, ε is strain.

The electrothermal performances of the papers were tested using a DC power (Keithley 2460, USA) supply of defined voltage, and the surface temperature of the papers was measured and captured by an infrared (IR) thermal imager (VarioCAM HD head 880, InfraTec, Germany). The photothermal performance of papers was studied with xenon lamp (CEL-HXF300-T3, China) irradiation to simulate sunlight.

**S2 Measurement of EMI Shielding Performance**

A N5227A (Keysight Technologies, USA) vector network analyzer was used to test the EMI SE value of the HMP paper in the X-band (8.2-12.4 GHz). The scattering parameters S11 and S21 were tested and used to calculate the reflection (R), transmission (T), and absorption (A) coefficients. At the same time, the total EMI shielding effectiveness (SE_T_), reflected EMI shielding effectiveness (SE_R_), absorbed EMI shielding effectiveness (SE_A_), and multiple internal reflected EMI shielding effectiveness (SE_M_) were calculated by the following formula:

$R=\left| S_{11} \right|^{2}$, $T=\left| S_{21} \right|^{2} (S2)$

$$R+A+T=1 (S3)$$

$$SE_{R}=-10\log\left( 1-R \right) (S4)$$

$$SE_{A}=-10\log\left( \frac{T}{1-R} \right) (S5)$$

$$SE_{T}=SE_{R}+SE_{A}+SE_{M} (S6)$$

If SE_T_ exceeds 10 dB, SE_M_ can be generally ignored [S1].

EMI shielding efficiency (%) expressed the ability to shield electromagnetic waves as a percentage. The calculation formula was as follows:

$$shielding efficiency\left( \% \right)=100-\left( \frac{1}{{10}^{\mathrm{SE}/{10}}} \right)\times100 (S7)$$

To fairly compare the effectiveness of EMI shielding materials, the specific shielding efficiency (SSE) and SSE/t expressions considering density and thickness were as follows:

$$SSE=EMI SE/density=dB \mathrm{cm}^{3} g^{-1} (S8)$$

$$SSE/t =SSE/thickness=dB \mathrm{cm}^{2}g^{-1} (S9)$$

According to the transmission line principle:

$$Z_{in}=Z_{0}\sqrt{\frac{\mu_{r}}{\varepsilon_{r}}}\tanh\left( 2\pi j\frac{fd\sqrt{\mu_{r}\varepsilon_{r}}}{c} \right) (S10)$$

where Z_in_ is the input impedance of the absorber, Z_0_ is the impedance of free space, *μ_r_* is the relative complex permeability, *ε_r_* is the complex permittivity, *f* is the frequency of microwaves, *d* is the thickness of the absorber, and *c* is the velocity of light.

**S3 Supplementary Fiugures and Tables**


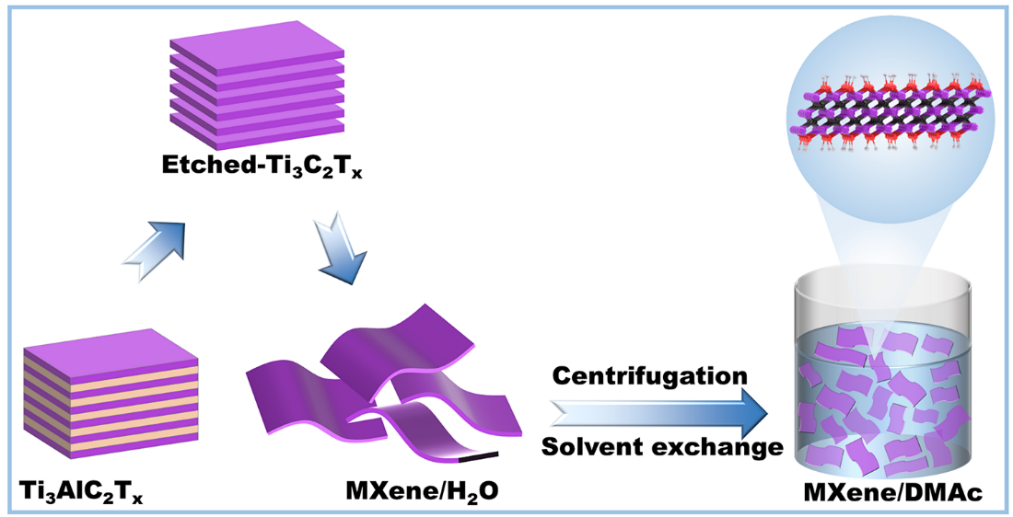


**Fig. S1** Schematic illustration of the fabrication of MXene/DMAc dispersion


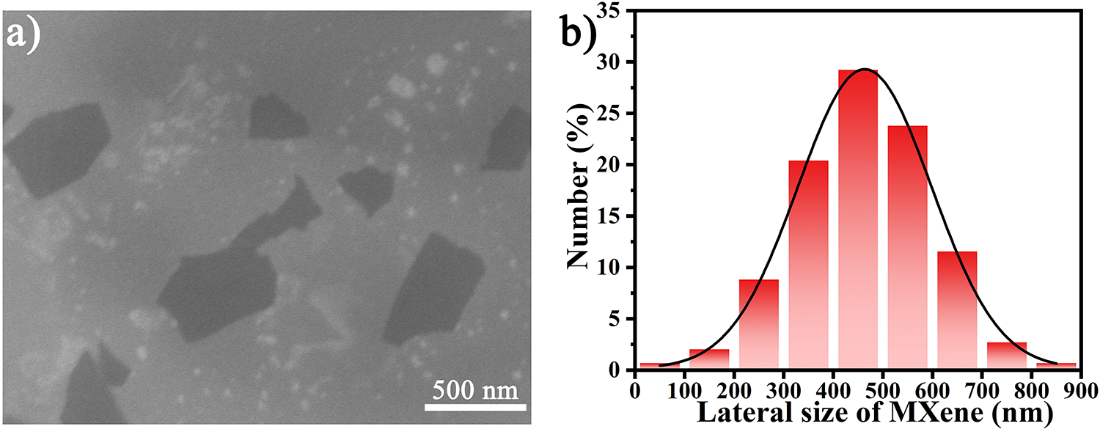


**Fig. S2** **a** SEM Morphology of the delaminated Ti_3_C_2_T_x_ MXene nanosheets. **b** Lateral size distribution of MXene nanosheets


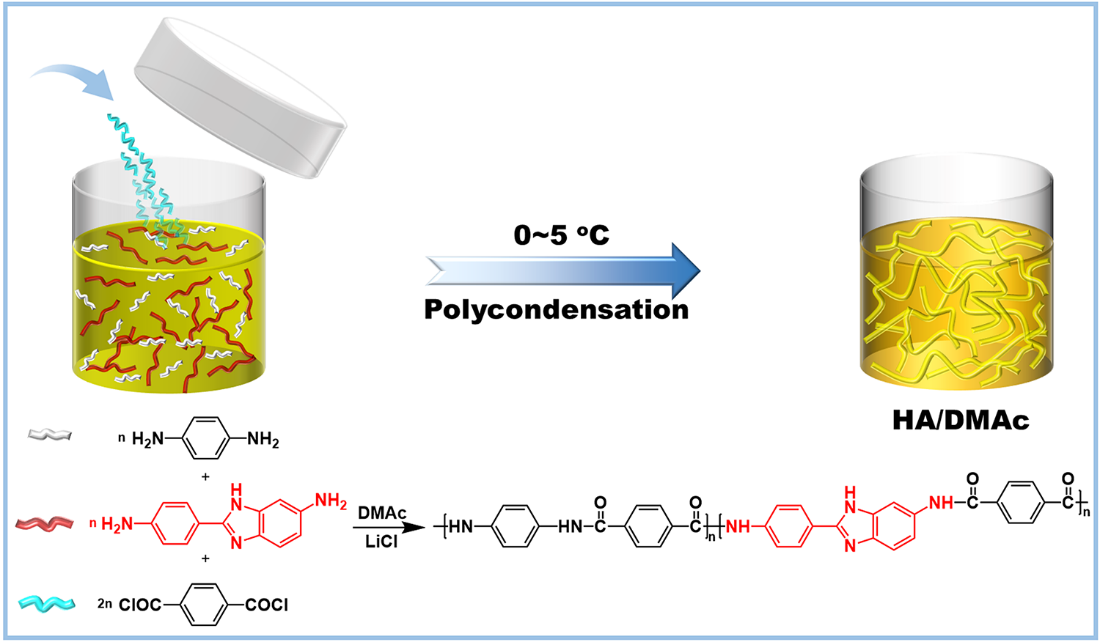


**Fig. S3** Schematic illustration of the polymerization of HA solution


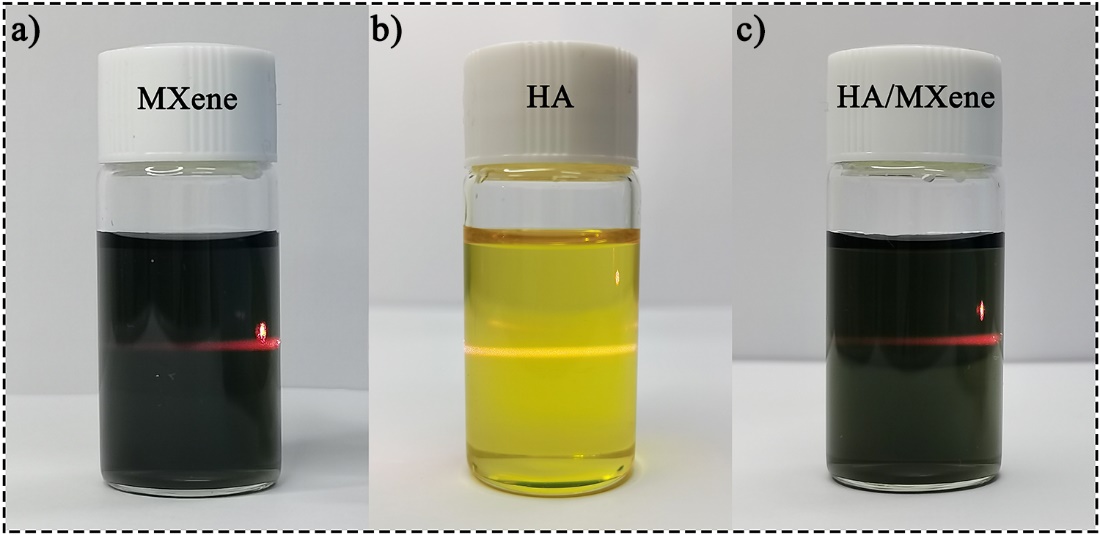


**Fig. S4** Tyndall effect of **a** MXene/DMAc, **b** HA/DMAc, and **c** HA/MXene/DMAc dispersion


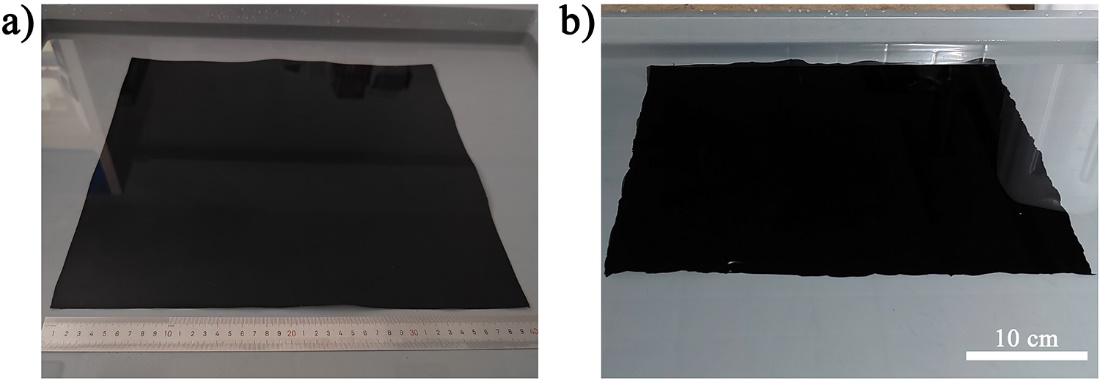


**Fig. S5** Digital image of the **a** HA/MXene and **b** HMP hydrogel


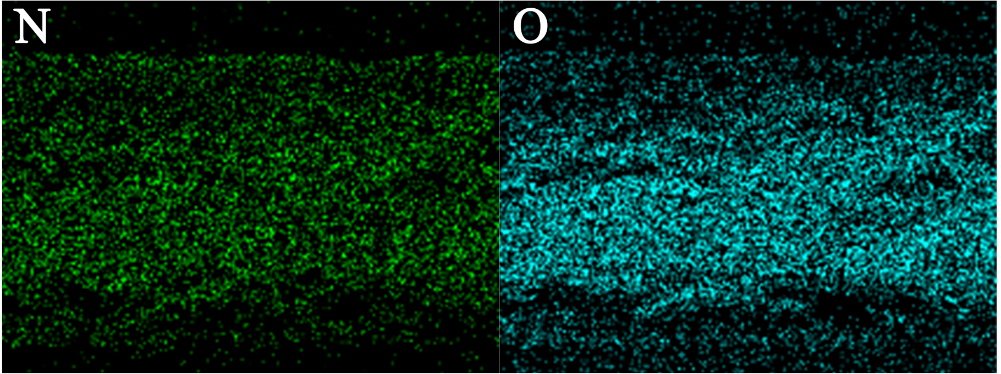


**Fig. S6** N- and O- element mappings of the HMP paper cross-section


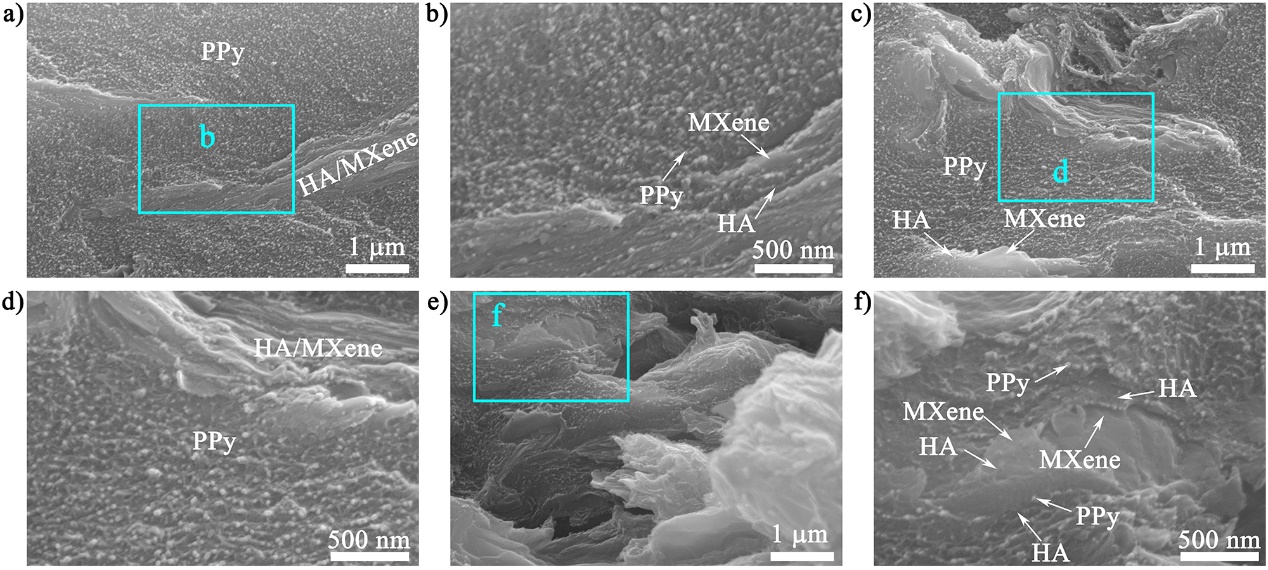


**Fig. S7 a, c, e** Cross-sectional SEM images of HMP nanocomposite paper. **b, d, f** Magnified images of a, c, and e, respectively


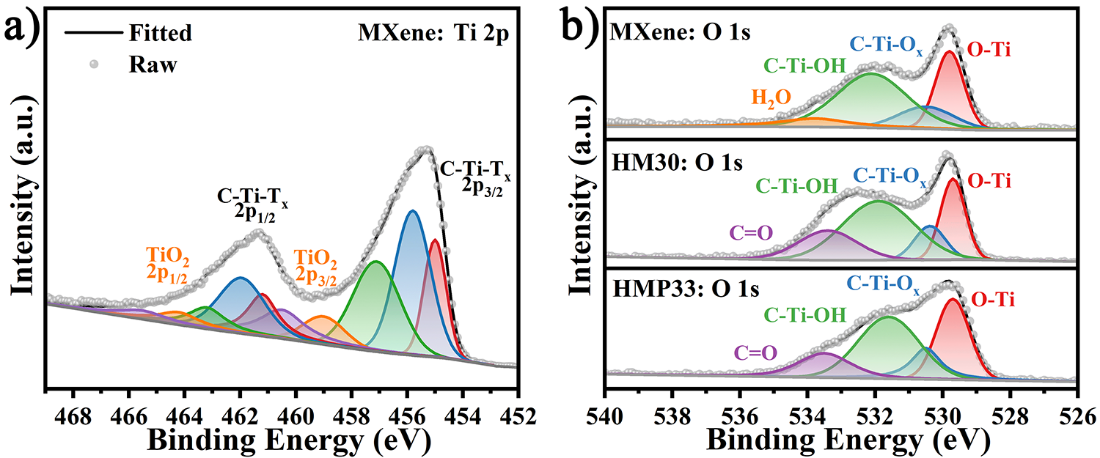


**Fig. S8 a** Ti 2p spectra of MXene paper. **b** O 1s spectra of MXene, MH30, and HMP33 papers


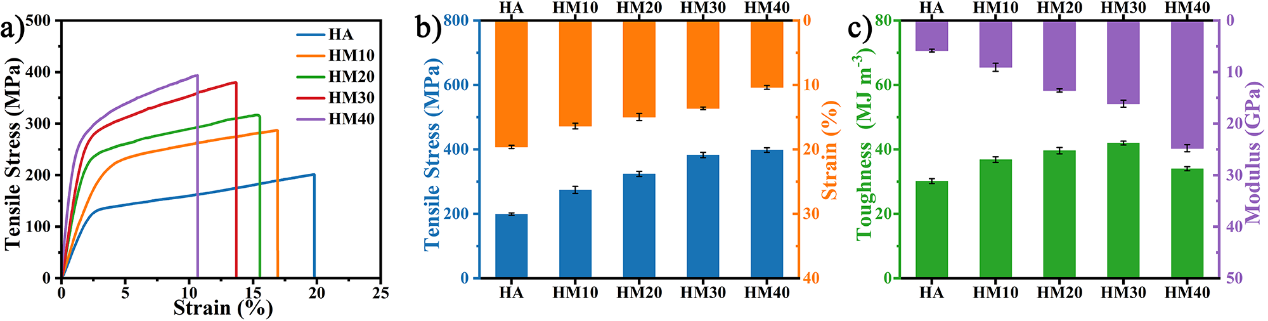


**Fig. S9 a** Typical stress-strain curves of HA/MXene papers with various MXene contents and corresponding **b** tensile strength, fracture strain, **c** toughness, and modulus


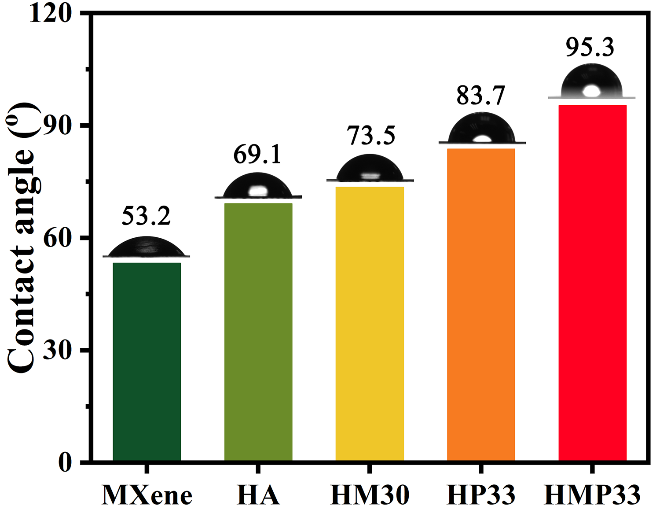


**Fig. S10** Water contact angle (CA) measurements for the MXene, HA, HM30, HP33, and HMP33 papers


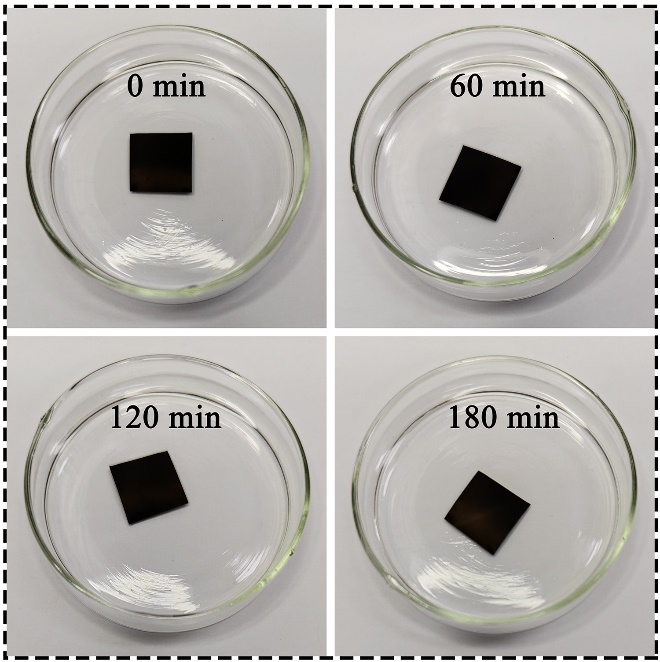


**Fig. S11** Optical images of the HMP33 paper during ultrasonication in water


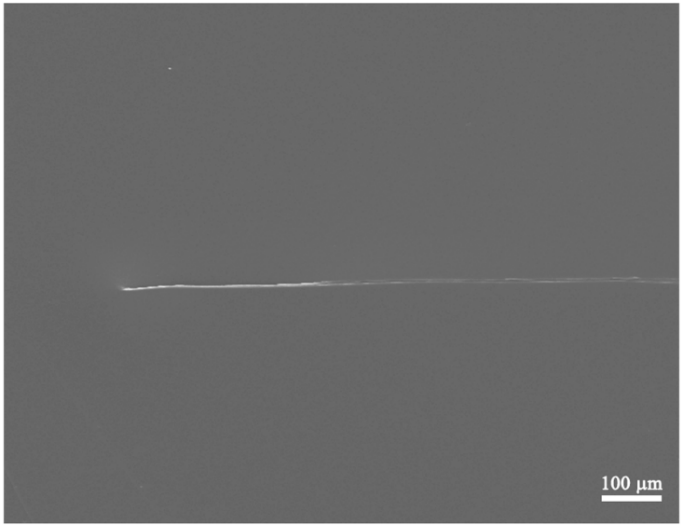


**Fig. S12** SEM image of fracture path of HA paper


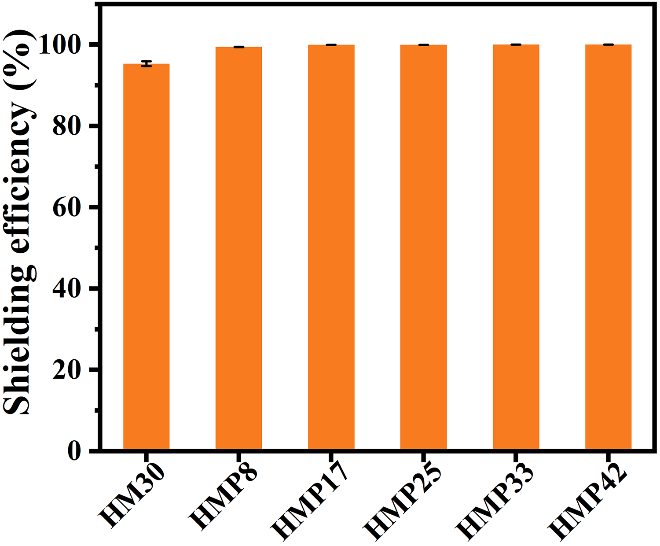


**Fig. S13** The shielding efficiencies of the HMP papers with different PPy loading


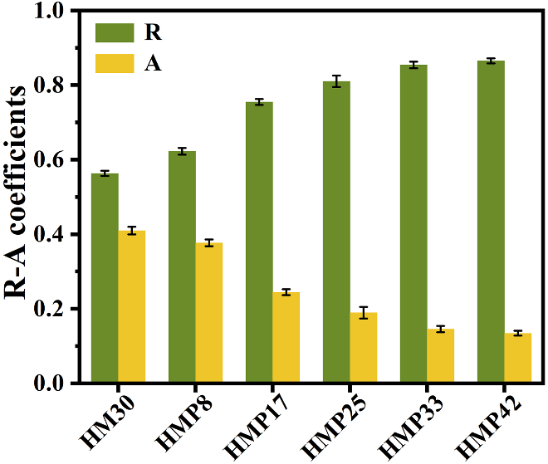


**Fig. S14** The average R and A values of the HMP nanocomposite paper in the X-band


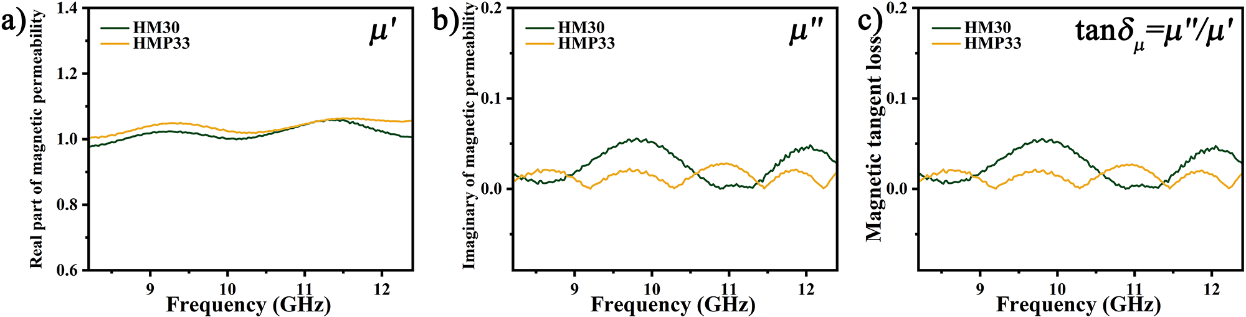


**Fig. S15** The **a** real part and **b** imaginary part of complex magnetic permeability, and **c** tan*δ_μ_* of the HM30 and HMP33


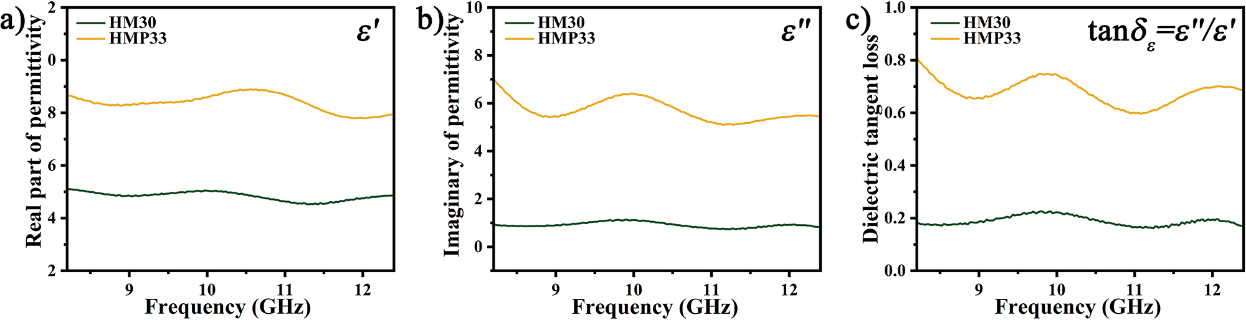


**Fig. S16 a** Real part and **b** imaginary part of complex permittivity, and **c** tan*δ_ε_* of the HM30 and HMP33


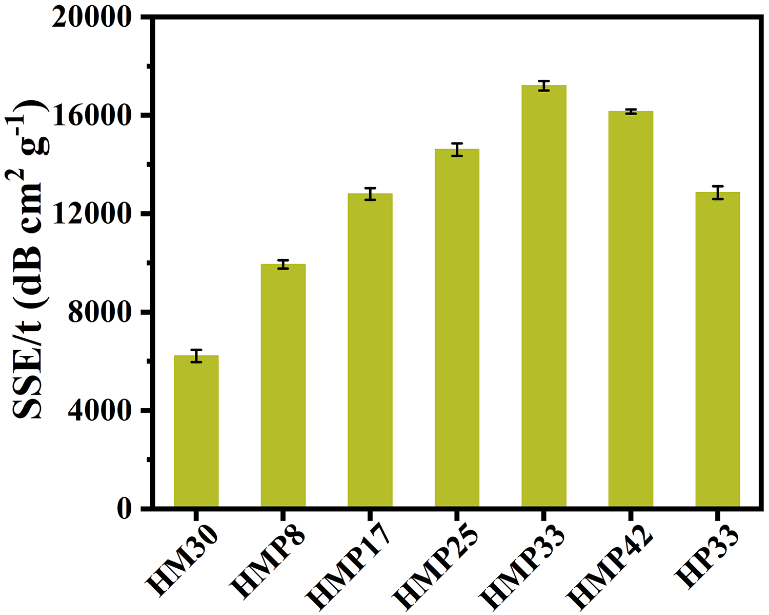


**Fig. S17** SSE/t of the HM30, HP33, and HMP papers


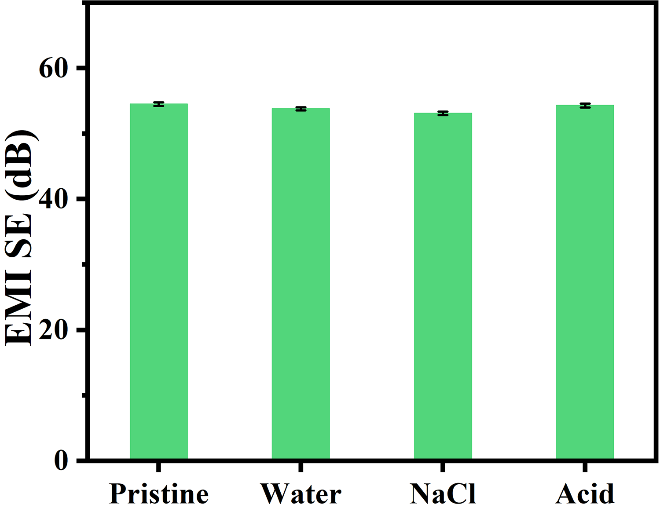


**Fig. S18** EMI SE of the HMP33 paper after soaking in various solvents for 7 days


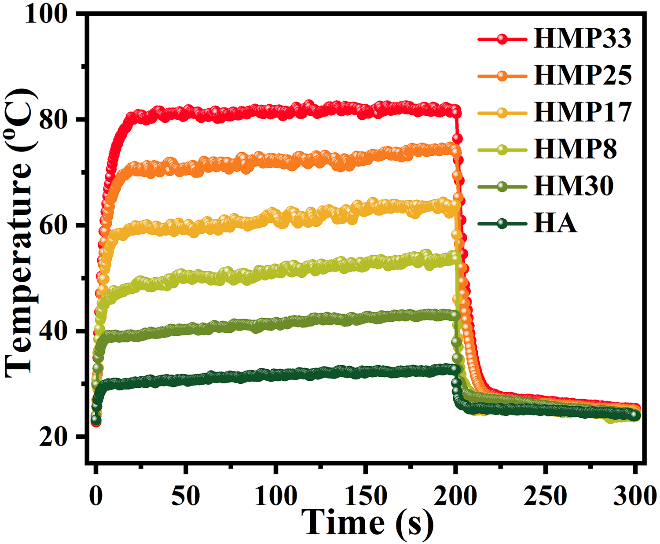


**Fig. S19** The photothermal curves of the papers with various PPy contents under the xenon lamp irradiation at a 100 mW·cm^-2^ light intensity


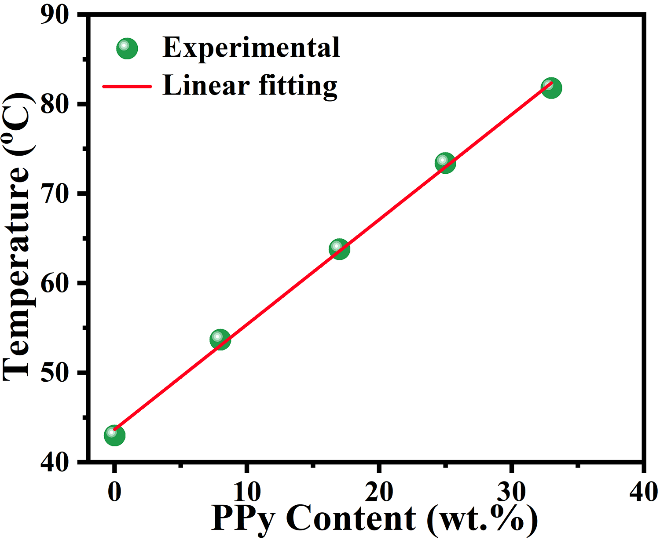


**Fig. S20** The equilibrium temperature of the HMP papers versus the PPy content

**Table S1** Tensile mechanical properties of various papers

| **Samples** | **Tensile stress/MPa** | **Strain/%** | **Toughness/MJ m^-3^** | **Modulus/GPa** |
| --- | --- | --- | --- | --- |
| HA | 199.05±3.85 | 19.60±0.25 | 30.16±0.78 | 5.82±0.26 |
| HM10 | 274.3±10.96 | 16.37±0.45 | 36.86±0.88 | 9.07±0.79 |
| HM20 | 324.06±7.88 | 14.97±0.55 | 39.63±0.99 | 13.55±0.32 |
| HM30 | 382.71±8.31 | 13.63±0.21 | 41.98±0.60 | 16.14±0.68 |
| HM40 | 397.93±7.16 | 10.37±0.29 | 34.01±0.62 | 24.78±0.70 |
| HMP8 | 364.68±9.02 | 15.33±0.50 | 44.93±1.42 | 12.98±0.12 |
| HMP17 | 348.87±12.09 | 16.86±0.34 | 47.83±0.86 | 9.96±0.45 |
| HMP25 | 332.33±9.07 | 19.45±0.51 | 51.41±1.36 | 8.29±0.25 |
| HMP33 | 309.67±5.69 | 22.38±0.48 | 57.63±0.81 | 7.64±0.28 |
| HMP42 | 215.57±9.08 | 14.99±0.34 | 29.38±1.11 | 7.04±0.06 |
| HP33 | 258.10±10.55 | 23.62±0.54 | 50.67±0.31 | 6.51±0.16 |

**Table S2** Comparison of mechanical properties of HMP nanocomposite papers with other EMI composite materials. For each reference, the maximum tensile stress and toughness are used to compare with this work

| **Types** | **Materials** | **Tensile stress**  **(MPa)** | **Strain**  **(%)** | **Toughness (MJ m^-3)^** | **Refs.** |
| --- | --- | --- | --- | --- | --- |
| Conductive polymer-based | PPy/GO-MP-CF-Epoxy | 308 | - | 3.2 | [S2] |
|  | PPy/CNF/graphene/PVA | 217.9 | 12.0 | 19.6 | [S3] |
|  | PANI/CNT | 232.3 | 11.5 | 20.1 | [S4] |
|  | PANI/Epoxy | 106.0 | 9.6 | 5.06 | [S5] |
|  | PANI/carbon-PTFE/GF | 21.5 | 12 | 17.5 | [S6] |
|  | PANI/Epoxy | 88.81 | 6.8 | 3.95 | [S7] |
|  | PEDOT:PSS/MXene | 38.6 | 0.28 | ~0.05 | [S8] |
|  | PEDOT:PSS-ANF/MXene | 155.9 | 20 | 19.4 | [S9] |
| MXene-based | MXene/ANF | 198.80 | 15.30 | 21.87 | [S10] |
|  | MXene/ANF | 177.9 | 13.3 | 13.7 | [S11] |
|  | MXene/ANF | 230.5 | 6.2 | 11.8 | [S12] |
|  | MXene/ANF | 232.0 | 8.2 | 13.4 | [S13] |
|  | MXene/ANF | 300.5 | 3.0 | 5.3 | [S14] |
|  | MXene@ANF | 243.04 | 9.05 | 12.21 | [S15] |
|  | MXene/Kevlar nanofiber | 101 | 4.6 | 2.6 | [S16] |
|  | CNF-MXene-TA | 275.4 | 5.8 | 10.2 | [S17] |
|  | MXene/CNF/PDA | 237.1 | 7.2 | 8.5 | [S18] |
|  | ANF-MXene/AgNW | 235.9 | 24.8 | ~20 | [S19] |
|  | MXene-xanthan | 121.09 | 6.31 | 4.43 | [S20] |
|  | MXene/TOCNF | 171.5 | 5.3 | 6.1 | [S21] |
|  | MXene/HNFs | 178.24 | 8.04 | 9.35 | [S22] |
|  | CNF@MXene | 112.5 | 4.3 | 2.7 | [S23] |
|  | MXene/CNF | 135.4 | 16.7 | 14.8 | [S24] |
|  | PDA/MXene | 309 | 3.5 | 4.4 | [S25] |
|  | GO/MXene/cellulose | 324 | 4.6 | 9.8 | [S26] |
|  | MXene/PU | 33.27 | 67.15 | 18.47 | [S27] |
|  | CNF/MXene | 114.4 | 2.8 | 2.6 | [S28] |
|  | CNTs/MXene/cellulose | 97.9 | 4.6 | 2.1 | [S29] |
|  | CNF/MXene@Ag | 78.5 | 4.6 | 3.0 | [S30] |
|  | CuNW/MXene/ANF | 206.55 | 10.92 | 19.97 | [S31] |
| Graphene-based | rGO/CA | 118.0 | 8.5 | 4.6 | [S32] |
|  | Graphene/ANF | 131.2 | 9.58 | 8.84 | [S33] |
|  | f-G/ANF | 158.9 | 16.0 | 16.5 | [S34] |
|  | rGO/PVA | 188.9 | 2.67 | 2.52 | [S35] |
|  | NFC/Graphene | 116.78 | 4.2 | 3.50 | [S36] |
|  | Graphene/rGO | 90 | 1.3 | 0.86 | [S37] |
| CNT-based | PDA@CNT | 49.2 | 4.4 | 1.3 | [S38] |
|  | PVP@CNT | 92.2 | 4.5 | 2.6 |  |
|  | PDA/PVP@CNT | 143.5 | 6.3 | 5.0 |  |
|  | CNT/Cellulose | 77.8 | 9.2 | 5.8 | [S39] |
|  | CNT/NR | 22.2 | 15.3 | 3.02 | [S40] |
|  | CNT/PEO&NFC/Fe_3_O_4_ | 36.03 | 19.1 | 2.98 | [S41] |
| This work | HMP8 | 364.68 | 15.33 | 44.93 |  |
|  | HMP17 | 348.87 | 16.86 | 47.83 |  |
|  | HMP25 | 332.33 | 19.45 | 51.41 |  |
|  | HMP33 | 309.67 | 22.38 | 57.63 |  |

**- No value available**

PPy: polypyrrole, GO: graphene oxide, MP: magnetite nanoparticles, CF: carbon fibers, CNF: cellulose nanofiber, PVA: Poly(vinyl alcohol), PANI: Polyaniline, CNT: carbon nanotube, PTFE: polytetrafluoroethylene, GF: glass fabric, PEDOT:PSS: poly(3,4-ethylenedioxythiophene):poly(styrenesulfonate), ANF: aramid nanofiber, TA: tannic acid, PDA: polydopamine, AgNW: silver nanowire, TOCNF: 2, 2, 6, 6-Tetramethyl-1piperidinyloxy (TEMPO) oxidized cellulose nanofibers, HNFs: holocellulose nanofibers, PU: polyurethane, CuNW: copper nanowire, rGO: reduced graphene oxide, CA: calcium alginate, f-G: fluorinated graphene, NFC: nanofibrillated cellulose, PVP: polyvinylpyrrolidone, NR: natural rubber, PEO: Polyethylene oxide.

**Table S3** The EMI shielding performance of HA, HM30, HP33, and HMP papers

| **Samples** | **Density (g cm^-3^)** | **Thickness (mm)** | **Electrical conductivity** | **EMI SE (dB)** | **SSE/t**  **(dB cm^2^ g^-1^)** |
| --- | --- | --- | --- | --- | --- |
| HA | 1.380 | 24.8 | 8.2×10^-11^ | 0.05 | 14.6 |
| HM30 | 1.611 | 13.3 | 7.3 | 13.3 | 6219.8 |
| HMP8 | 1.524 | 14.9 | 105.6 | 22.6 | 9940.9 |
| HMP17 | 1.398 | 19.3 | 1848.8 | 34.6 | 12806.4 |
| HMP25 | 1.306 | 22.5 | 8355.7 | 42.9 | 14607.2 |
| HMP33 | 1.237 | 25.4 | 17386.2 | 54.1 | 17204.7 |
| HMP42 | 1.194 | 30.9 | 20914.0 | 59.6 | 16155.9 |
| HP33 | 1.209 | 25.6 | 6297.0 | 39.8 | 12856.1 |

**Table S4** Comparison of the EMI shielding performance of the HMP nanocomposite papers with other EMI materials

| **Types** | **Materials** | **Thickness (mm)** | **EMI SE (dB)** | **SSE/t**  **(****dB cm^2^ g^-1^)** | **Refs.** |
| --- | --- | --- | --- | --- | --- |
| Metal-based | Cu Foil | 0.01 | 70 | 7812 | [S42] |
|  | CF/PC/Ni | 0.31 | 72.7 | 1376.1 | [S43] |
|  | AgNW/cellulose | 0.16 | 48.6 | 5585.0 | [S44] |
|  | PP/PDA/Ag | 0.166 | 25.01 | 6097.56 | [S45] |
|  | Ag/magnetic CNT/PP | 0.53 | 61.1 | 2811.78 | [S46] |
| MXene-based | Ti_3_C_2_T_x_/TOCNF | 0.035 | 39.6 | 4750 | [S21] |
|  | Ti_3_C_2_T_x_/CNF | 0.047 | 24.0 | 2647 | [S24] |
|  | CNT/MXene/Cellulose | 0.038 | 23.4 | 5219 | [S29] |
|  | PVA/MXene | 0.027 | 44.4 | 9343 | [S47] |
|  | CuNW/MXene/ANF | 0.043 | 46.67 | 9120.6 | [S31] |
|  | HNFs/MXene | 0.036 | 32.09 | 8501.84 | [S22] |
|  | CNF/GNPs/MXene | 0.0385 | 33.74 | 5332.62 | [S48] |
|  | MXene/MMT | 0.03 | 57.8 | 8381.5 | [S23] |
|  | Ti_3_C_2_T_x_/PNFs | 0.0279 | 43 | 7972 | [S49] |
|  | CNF@MXene | 0.035 | 39.6 | 7029 | [S23] |
|  | MXene/ANF | 0.01097 | 21 | 9555.7 | [S14] |
|  | MXene/ANF | 0.037 | 48.0 | 13188.2 | [S10] |
| PPy-based | ANF@PPy | 0.07576 | 41.69 | 2762.5 | [S50] |
|  | PAN@Ag@PDA@PPy | 0.16 | 23.81 | 6764.2 | [S51] |
|  | PPy/CF | 0.45 | 23.9 | 513 | [S52] |
|  | TiO_2_/SiO_2_@PPy@rGO | 0.26 | 32.0 | 13829 | [S53] |
|  | MNPs/TPU/PPy | 0.2 | 26.3 | 1563.17 | [S54] |
| Graphene-based | Graphene/PDMS | 1 | 20 | 3333 | [S55] |
|  | Graphene/PEDOT:PSS | 1.5 | 91.9 | 8040 | [S56] |
|  | Graphene/B_4_C | 1.5 | 40 | 100 | [S57] |
|  | Graphene/P(St-BA) | 0.05 | 21.5 | 2663 | [S58] |
|  | Graphene/PS | 0.25 | 45.1 | 692 | [S59] |
|  | Graphene/PE | 2 | 32.4 | 318 | [S60] |
| CNT-based | CNT/MFC | 0.036 | 26.67 | 9944 | [S61] |
|  | CNF/MWCNT | 0.15 | 46.4 | 4017.3 | [S62] |
|  | MWCNT/WPU | 1.1 | 35 | 2143 | [S63] |
|  | CNT/BC | 0.036 | 24.01 | 9074 | [S64] |
|  | CNT/epoxy | 1.8 | 34.5 | 535 | [S65] |
|  | CNT/NR | 0.05 | 31.9 | 8504 | [S66] |
| This work | HMP8 | 0.0149 | 22.57 | 9940.9 |  |
|  | HMP17 | 0.0193 | 34.55 | 12806.4 |  |
|  | HMP25 | 0.0225 | 42.923 | 14607.2 |  |
|  | HMP33 | 0.0254 | 54.06 | 17204.7 |  |
|  | HMP42 | 0.0309 | 59.61 | 16155.9 |  |

CF: nonwoven carbon fabric, PC: polycarbonate, AgNW: silver nanowire, PP: polypropylene, PDA: polydopamine, CNT: carbon nanotube, TOCNF: 2, 2, 6, 6-Tetramethyl-1piperidinyloxy (TEMPO) oxidized cellulose nanofibers, CNF: cellulose nanofiber, PVA: polyvinyl alcohol, CuNW: copper nanowire, ANF: aramid nanofiber, HNFs: holocellulose nanofibers, GNPs: graphene nanoplatelets, MMT: montmorillonite, PNFs: poly(p‐phenylene‐2,6benzobisoxazole) (PBO) nanofibers, PAN: polyacrylonitrile, CF: chrome-tanned collagen fiber, rGO: reduced graphene oxide, MNPs: magnetic nanoparticles, TPU: thermoplastic polyurethane, PDMS: poly(dimethyl siloxane), PEDOT:PSS: poly(3,4-ethylenedioxythiophene):poly(styrenesulfonate), P(St-BA): poly(styrene-butyl acrylate) latex, PS: polystyrene, PE: polyethylene, MFC: microfibrillated cellulose, MWCNT: multi-walled carbon nanotube, WPU: water-borne polyurethane, BC: bacterial cellulose, NR: natural rubber.

**Supplementary References**

1. Y. Sun, X. Han, P. Guo, Z. Chai, J. Yue et al., Slippery graphene-bridging liquid metal layered heterostructure nanocomposite for stable high-performance electromagnetic interference shielding. ACS Nano **17**(13), 12616-12628 (2023). <https://doi.org/10.1021/acsnano.3c02975>
2. R. Kanwal, M. F. Maqsood, M. A. Raza, A. Inam, M. Waris et al., Polypyrrole coated carbon fiber/magnetite/graphene oxide reinforced hybrid epoxy composites for high strength and electromagnetic interference shielding. Mater. Today Commun. **38**, 107684 (2024). <https://doi.org/10.1016/j.mtcomm.2023.107684>
3. Z. Wang, L. Mo, S. Zhao, J. Li, S Zhang et al., Mechanically robust nacre-mimetic framework constructed polypyrrole-doped graphene/nanofiber nanocomposites with improved thermal electrical properties. Mater. Des. **155**, 278-287 (2018). <https://doi.org/10.1016/j.matdes.2018.06.004>
4. J. Huang, X. Liu, Y. Du, Highly efficient and wearable thermoelectric composites based on carbon nanotube film/polyaniline. J. Materiomics **10**(1), 173-178 (2024). <https://doi.org/10.1016/j.jmat.2023.04.014>
5. J. Guo, Z. Chen, X. Xu, X. Li, H. Liu et al., Enhanced electromagnetic wave absorption of engineered epoxy nanocomposites with the assistance of polyaniline fillers. Adv. Compos. Hybrid. Mater. **5**(3), 1769-1777 (2022). <https://doi.org/10.1007/s42114-022-00417-2>
6. B. Zhang, Y. Jiang, Simultaneous reinforcing and toughening of PTFE/glass fabric composites based on polyaniline/carbon microcapsule network. Nano-Micro Lett. **13**(11), 1615-1620 (2018). <https://doi.org/10.1049/mnl.2018.5118>
7. J. Guo, J. Long, D. Ding, Q. Wang, Y. Shan et al., Significantly enhanced mechanical and electrical properties of epoxy nanocomposites reinforced with low loading of polyaniline nanoparticles. RSC Adv. **6**(25), 21187-21192 (2016). <https://doi.org/10.1039/c5ra25210e>
8. Y.-J. Wan, X.-M. Li, P.-L. Zhu, R. Sun, C.-P. Wong et al., Lightweight, flexible MXene/polymer film with simultaneously excellent mechanical property and high-performance electromagnetic interference shielding. Compos. Part. A-Appl. Sci. Manuf. **130**, 105764 (2020). <https://doi.org/10.1016/j.compositesa.2020.105764>
9. J. Li, Y. Wen, Z. Xiao, S. Wang, L. Zhong et al., Holey reduced graphene oxide scaffolded heterocyclic aramid fibers with enhanced mechanical performance. Adv. Funct. Mater. **32**(42), 2200937 (2022). <https://doi.org/10.1002/adfm.202200937>
10. J. Wang, X. Ma, J. Zhou, F. Du, C. Teng, Bioinspired, high-strength, and flexible MXene/aramid fiber for electromagnetic interference shielding papers with Joule heating performance. ACS Nano **16**(4), 6700-6711 (2022). <https://doi.org/10.1021/acsnano.2c01323>
11. D. Hu, S. Wang, C. Zhang, P. Yi, P. Jiang, et al., Ultrathin MXene-aramid nanofiber electromagnetic interference shielding films with tactile sensing ability withstanding harsh temperatures. Nano Res. **14**(8), 2837-2845 (2021). <https://doi.org/10.1007/s12274-021-3297-z>
12. Z. Cheng, Y. Cao, R. Wang, X. Liu, F. Fan et al., Multifunctional MXene-based composite films with simultaneous terahertz/gigahertz wave shielding performance for future 6G communication. J. Mater. Chem. A **11**(11), 5593-5605 (2023). <https://doi.org/10.1039/d2ta09879b>
13. C. Weng, T. Xing, H. Jin, G. Wang, Z. Dai et al., Mechanically robust ANF/MXene composite films with tunable electromagnetic interference shielding performance. Compos. Part. A-Appl. Sci. Manuf. **135**, 105927 (2020). <https://doi.org/10.1016/j.compositesa.2020.105927>
14. C. Lei, Y. Zhang, D. Liu, K. Wu, Q. Fu, Metal-level robust, folding endurance, and highly temperature-stable MXene-based film with engineered aramid nanofiber for extreme-condition electromagnetic interference shielding applications. ACS Appl. Mater. Interfaces **12**(23), 26485-26495 (2020). <https://doi.org/10.1021/acsami.0c07387>
15. J. Lu, L. Cheng, C. Liao, P. Jia, L. Song et al., Ultrathin and mechanically robust mussel byssus‐inspired MXene@aramid nanofibers materials with superior endurance in harsh environments for tunable emi shielding performance. Adv. Mater. Interfaces **9**(5), 2101359 (2022). <https://doi.org/10.1002/admi.202101359>
16. Z. Zhang, S. Yang, P. Zhang, J. Zhang, G. Chen et al., Mechanically strong MXene/kevlar nanofiber composite membranes as high-performance nanofluidic osmotic power generators. Nat. Commun. **10**(1), 2920 (2019). <https://doi.org/10.1038/s41467-019-10885-8>
17. J. Wei, S. Jia, J. Wei, C. Ma, Z. Shao, Tough and multifunctional composite film actuators based on cellulose nanofibers toward smart wearables. ACS Appl. Mater. Interfaces **13**(32), 38700-38711 (2021). <https://doi.org/10.1021/acsami.1c09653>
18. J. Cao, Z. Zhou, Q. Song, K. Chen, G. Su et al., Ultrarobust Ti_3_C_2_T_x_ MXene-based soft actuators via bamboo-inspired mesoscale assembly of hybrid nanostructures. ACS Nano. **14**(6), 7055-7065 (2020). <https://doi.org/10.1021/acsnano.0c01779>
19. Z. Ma, S. Kang, J. Ma, L. Shao, Y. Zhang et al., Ultraflexible and mechanically strong double-layered aramid nanofiber-Ti_3_C_2_T_x_ MXene/silver nanowire nanocomposite papers for high-performance electromagnetic interference shielding. ACS Nano. **14**(7), 8368-8382 (2020). <https://doi.org/10.1021/acsnano.0c02401>
20. Y. Sun, R. Ding, S. Y. Hong, J. Lee, Y.-K. Seo et al., MXene-xanthan nanocomposite films with layered microstructure for electromagnetic interference shielding and Joule heating. Chem. Eng. J. **410**, 128348 (2021). <https://doi.org/10.1016/j.cej.2020.128348>
21. Z. Zhan, Q. Song, Z. Zhou, C. Lu, Ultrastrong and conductive MXene/cellulose nanofiber films enhanced by hierarchical nano-architecture and interfacial interaction for flexible electromagnetic interference shielding. J. Mater. Chem. C **7**(32), 9820-9829 (2019). <https://doi.org/10.1039/c9tc03309b>
22. J. Rao, Q. Ding, Z. Lv, D. Sun, B. Lüet al., Strong holocellulose-based nanopaper with a sandwich-like structure for effective electromagnetic shielding. ACS Sustainable Chem. Eng. **10**(34), 11396-11405 (2022). <https://doi.org/10.1021/acssuschemeng.2c03474>
23. B. Zhou, Z. Zhang, Y. Li, G. Han, Y. Feng et al., Flexible, robust, and multifunctional electromagnetic interference shielding film with alternating cellulose nanofiber and MXene layers. ACS Appl. Mater. Interfaces **12**(4), 4895-4905 (2020). <https://doi.org/10.1021/acsami.9b19768>
24. W. T. Cao, F. F. Chen, Y. J. Zhu, Y. G. Zhang, Y. Y. Jiang et al., Binary strengthening and toughening of MXene/cellulose nanofiber composite paper with nacre-inspired structure and superior electromagnetic interference shielding properties. ACS Nano. **12**(5), 4583-4593 (2018). <https://doi.org/10.1021/acsnano.8b00997>
25. G. S. Lee, T. Yun, H. Kim, I. H. Kim, J. Choi et al., Mussel inspired highly aligned Ti_3_C_2_T_x_ MXene film with synergistic enhancement of mechanical strength and ambient stability. ACS Nano. **14**(9), 11722-11732 (2020). <https://doi.org/10.1021/acsnano.0c04411>
26. B. Li, N. Wu, Y. Yang, F. Pan, C. Wang et al., Graphene oxide‐assisted multiple cross‐linking of MXene for large‐area, high‐strength, oxidation‐resistant, and multifunctional films. Adv. Funct. Mater. **33**(11), 2213357 (2022). <https://doi.org/10.1002/adfm.202213357>
27. J. Zhou, D. Shi, Y. Wang, M. Chen, W. Dong, Bioinspired MXene/polyurethane plastic films with exceptional flexibility and toughness for electromagnetic interference shielding. Mater. Res. Bull. **154**, 111939 (2022). <https://doi.org/10.1016/j.materresbull.2022.111939>
28. E. Jiao, K. Wu, Y. Liu, M. Lu, Z. Hu et al., Ultrarobust MXene-based laminated paper with excellent thermal conductivity and flame retardancy. Compos. Part. A-Appl. Sci. Manuf. **146**, 106417 (2021). <https://doi.org/10.1016/j.compositesa.2021.106417>
29. W. Cao, C. Ma, S. Tan, M. Ma, P. Wan et al., Ultrathin and flexible CNTs/MXene/cellulose nanofibrils composite paper for electromagnetic interference shielding. Nano-Micro Lett. **11**(1), 72 (2019). <https://doi.org/10.1007/s40820-019-0304-y>
30. E. Jiao, K. Wu, Y. Liu, M. Lu, H. Zhang et al., Robust bioinspired MXene-based flexible films with excellent thermal conductivity and photothermal properties. Compos. Part. A-Appl. Sci. Manuf. **143**, 106290 (2021). <https://doi.org/10.1016/j.compositesa.2021.106290>
31. F. Jia, J. Dong, X. Dai, Y. Liu, H. Wang et al., Robust, flexible, and stable CuNWs/MXene/ANFs hybrid film constructed by structural assemble strategy for efficient EMI shielding. Chem. Eng. J. **452**, 139395 (2023). <https://doi.org/10.1016/j.cej.2022.139395>
32. L.-C. Jia, W.-J. Sun, C.-G. Zhou, D.-X. Yan, Q.-C. Zhang et al., Integrated strength and toughness in graphene/calcium alginate films for highly efficient electromagnetic interference shielding. J. Mater. Chem. C **6**, 9166 (2018). <https://doi.org/10.1039/C8TC03151G>
33. L. Su, X. Ma, J. Wang, R. Zhai, C. Song et al., High-strength, flexible and superhydrophobic graphene/aramid nanofiber nanocomposite films for electromagnetic interference shielding application. Ceram. Int **48**(18), 26013-26021 (2022). <https://doi.org/10.1016/j.ceramint.2022.05.281>
34. L.-H. Zhao, Y.-F. Jin, Z.-G. Wang, J.-W. Ren, L.-C. Jia et al., Highly thermally conductive fluorinated graphene/aramid nanofiber films with superior mechanical properties and thermostability. Ind. Eng. Chem. Res. **60**(23), 8451-8459 (2021). <https://doi.org/10.1021/acs.iecr.1c01260>
35. Y. Q. Li, T. Yu, T. Y. Yang, L. X. Zheng, K. Liao, Bio‐inspired nacre‐like composite films based on graphene with superior mechanical, electrical, and biocompatible properties. Adv. Mater. **24**(25), 3426-3431 (2012). <https://doi.org/10.1002/adma.201200452>
36. N. Song, S. Cui, D. Jiao, X. Hou, P. Ding et al., Layered nanofibrillated cellulose hybrid films as flexible lateral heat spreaders: The effect of graphene defect. Carbon. **115**, 338-346 (2017). <https://doi.org/10.1016/j.carbon.2017.01.017>
37. S. Wang, X. Sun, F. Xu, M. Yang, W. Yin et al., Strong yet tough graphene/graphene oxide hybrid films. Carbon. **179**, 469-476 (2021). <https://doi.org/10.1016/j.carbon.2021.04.052>
38. S. Qu, X. Jiang, Q. Li, L. Gao, G. Zhou et al., Developing strong and tough carbon nanotube films by a proper dispersing strategy and enhanced interfacial interactions. Carbon. **149**, 117-124 (2019). <https://doi.org/10.1016/j.carbon.2019.04.033>
39. H.-D. Huang, C.-Y. Liu, L.-Q. Zhang, G.-J. Zhong, Z.-M. Li, Simultaneous reinforcement and toughening of carbon nanotube/cellulose conductive nanocomposite films by interfacial hydrogen bonding. ACS Sustainable Chem. Eng. **3**(2), 317-324 (2015). <https://doi.org/10.1021/sc500681v>
40. L.-C. Jia, M.-Z. Li, D.-X. Yan, C.-H. Cui, H.-Y. Wu et al., A strong and tough polymer-carbon nanotube film for flexible and efficient electromagnetic interference shielding. J. Mater. Chem. C **5**(35), 8944-8951 (2017). <https://doi.org/10.1039/c7tc02259j>
41. Y. Li, B. Xue, S. Yang, Z. Cheng , L. Xie et al., Flexible multilayered films consisting of alternating nanofibrillated cellulose/Fe_3_O_4_ and carbon nanotube/polyethylene oxide layers for electromagnetic interference shielding. Chem. Eng. J. **410**, 128356 (2021). <https://doi.org/10.1016/j.cej.2020.128356>
42. F. Shahzad, M. Alhabeb, C. B. Hatter, B. Anasori, S. M. Hong et al., Electromagnetic interference shielding with 2D transition metal. Science. **353**(6304), 1137-1140 (2016). <https://doi.org/10.1126/science.aag2421>
43. D. Xing, L. Lu, K. S. Teh, Z. Wan, Y. Xie et al., Highly flexible and ultra-thin ni-plated carbon-fabric/polycarbonate film for enhanced electromagnetic interference shielding. Carbon. **132**(32-41 (2018). <https://doi.org/10.1016/j.carbon.2018.02.001>
44. T. W. Lee, S. E. Lee, Y. G. Jeong, Highly effective electromagnetic interference shielding materials based on silver nanowire/cellulose papers. ACS Appl. Mater. Interfaces **8**(20), 13123-13132 (2016). <https://doi.org/10.1021/acsami.6b02218>
45. T. T. Li, X. Wang, Y. Wang, B. C. Shiu, H. K. Peng et al., Silver‐coated conductive composite fabric with flexible, anti‐flaming for electromagnetic interference shielding. J. Appl. Polym. Sci. **139**(13), 51875 (2021). <https://doi.org/10.1002/app.51875>
46. L. Hu, Z. Kang, Enhanced flexible polypropylene fabric with silver/magnetic carbon nanotubes coatings for electromagnetic interference shielding. Appl. Surf. Sci. **568**, 150845 (2021). <https://doi.org/10.1016/j.apsusc.2021.150845>
47. X. Jin, J. Wang, L. Dai, X. Liu, L. Li et al., Flame-retardant poly(vinyl alcohol)/MXene multilayered films with outstanding electromagnetic interference shielding and thermal conductive performances. Chem. Eng. J. **380**, 122475 (2020). <https://doi.org/10.1016/j.cej.2019.122475>
48. Q. Chu, H. Lin, M. Ma, S. Chen, Y. Shi et al., Cellulose nanofiber/graphene nanoplatelet/MXene nanocomposites for enhanced electromagnetic shielding and high in-plane thermal conductivity. ACS Appl. Nano Mater. **5**(5), 7217-7227 (2022). <https://doi.org/10.1021/acsanm.2c01126>
49. Y. Huo, D. Guo, J. Yang, Y. Chang, B. Wang et al., Multifunctional bacterial cellulose nanofibers/polypyrrole (PPy) composite films for Joule heating and electromagnetic interference shielding. ACS Appl. Electron. Mater. **4**(5), 2552-2560 (2022). <https://doi.org/10.1021/acsaelm.2c00316>
50. J. Zhou, S. Thaiboonrod, J. Fang, S. Cao, M. Miao et al., In-situ growth of polypyrrole on aramid nanofibers for electromagnetic interference shielding films with high stability. Nano Res. **15**(9), 8536-8545 (2022). <https://doi.org/10.1007/s12274-022-4628-4>
51. Y. Wang, H.-K. Peng, T.-T. Li, B.-C. Shiu, X. Zhang et al., Layer-by-layer assembly of low-temperature in-situ polymerized pyrrole coated nanofiber membrane for high-efficiency electromagnetic interference shielding. Prog. Org. Coating **147**, 105861 (2020). <https://doi.org/10.1016/j.porgcoat.2020.105861>
52. J. Zhang, Z. Yan, X. Liu, Y. Zhang, H. Zou et al., Conductive skeleton-heterostructure composites based on chrome shavings for enhanced electromagnetic interference shielding. ACS Appl. Mater. Interfaces **12**(47), 53076-53087 (2020). <https://doi.org/10.1021/acsami.0c14300>
53. L. Huang, J. Li, Y. Li, X. He, Y. Yuan, Lightweight and flexible hybrid film based on delicate design of electrospun nanofibers for high-performance electromagnetic interference shielding. Nanoscale. **11**(17), 8616-8625 (2019). <https://doi.org/10.1039/c9nr02102g>
54. W. Zhao, B. Zhao, Z. Wu, K. Pei, Y. Qian et al., Dopant engineering of flexible MNPs/TPU/PPy core-shell films for controllable electromagnetic interference shielding. ACS Appl. Mater. Interfaces **15**(23), 28410-28420 (2023). <https://doi.org/10.1021/acsami.3c02454>
55. L. Wei, W. Zhang, J. Ma, S.-L. Bai, Y. Ren et al., π-π stacking interface design for improving the strength and electromagnetic interference shielding of ultrathin and flexible water-borne polymer/sulfonated graphene composites. Carbon. **149**, 679-692 (2019). <https://doi.org/10.1016/j.carbon.2019.04.058>
56. D.-X. Yan, H. Pang, B. Li, R. Vajtai, L. Xu et al., Structured reduced graphene oxide/polymer composites for ultra-efficient electromagnetic interference shielding. Adv. Funct. Mater. **25**(4), 559-566 (2015). <https://doi.org/10.1002/adfm.201403809>
57. J. Jing, Y. Xiong, S. Shi, H. Pei, Y. Chen et al., Facile fabrication of lightweight porous FDM-printed polyethylene/graphene nanocomposites with enhanced interfacial strength for electromagnetic interference shielding. Compos. Sci. Technol. **207**(108732 (2021). <https://doi.org/10.1016/j.compscitech.2021.108732>
58. Z. Chen, C. Xu, C. Ma, W. Ren, H. M. Cheng, Lightweight and flexible graphene foam composites for high-performance electromagnetic interference shielding. Adv. Mater. **25**(9), 1296-1300 (2013). <https://doi.org/10.1002/adma.201204196>
59. Y. Wu, Z. Wang, X. Liu, X. Shen, Q. Zheng et al., Ultralight graphene foam/conductive polymer composites for exceptional electromagnetic interference shielding. ACS Appl. Mater. Interfaces **9**(10), 9059-9069 (2017). <https://doi.org/10.1021/acsami.7b01017>
60. Y. Q. Tan, H. Luo, X. S. Zhou, S. M. Peng, H. B. Zhang, Boron carbide composites with highly aligned graphene nanoplatelets: Light-weight and efficient electromagnetic interference shielding materials at high temperatures. RSC Adv. **8**(69), 39314-39320 (2018). <https://doi.org/10.1039/c8ra07351a>
61. M. Fan, R. Chen, Y. Lu, R. Liu, Y. Ma et al., Flexible microfibrillated cellulose/carbon nanotube multilayered composite films with electromagnetic interference shielding and thermal conductivity. Compos. Commun. **35**, 101293 (2022). <https://doi.org/10.1016/j.coco.2022.101293>
62. H. Zhang, X. Sun, Z. Heng, Y. Chen, H. Zou et al., Robust and flexible cellulose nanofiber/multiwalled carbon nanotube film for high-performance electromagnetic interference shielding. Ind. Eng. Chem. Res. **57**(50), 17152-17160 (2018). <https://doi.org/10.1021/acs.iecr.8b04573>
63. Z. Zeng, H. Jin, M. Chen, W. Li, L. Zhou et al., Lightweight and anisotropic porous MWCNT/WPU composites for ultrahigh performance electromagnetic interference shielding. Adv. Funct. Mater. **26**(2), 303-310 (2016). <https://doi.org/10.1002/adfm.201503579>
64. M. Fan, X. Xia, S. Li, R. Zhang, L. Wu et al., Sustainable bacterial cellulose reinforced carbon nanotube buckypaper and its multifunctionality for electromagnetic interference shielding, Joule heating and humidity sensing. Chem. Eng. J. **441**, 136103 (2022). <https://doi.org/10.1016/j.cej.2022.136103>
65. Y.-J. Mao, L. Xu, H. Lin, J. Li, D.-X. Yan et al., Effective electromagnetic interference shielding properties of micro-truss structured CNT/epoxy composites fabricated based on visible light processing. Compos. Sci. Technol. **221**, 109296 (2022). <https://doi.org/10.1016/j.compscitech.2022.109296>
66. M. Fan, S. Li, L. Wu, L. Li, M. Qu et al., Natural rubber toughened carbon nanotube buckypaper and its multifunctionality in electromagnetic interference shielding, thermal conductivity, Joule heating and triboelectric nanogenerators. Chem. Eng. J. **433**, 133499 (2022). <https://doi.org/10.1016/j.cej.2021.133499>
